# *aroA*-Deficient *Salmonella enterica* Serovar Typhimurium Is More Than a Metabolically Attenuated Mutant

**DOI:** 10.1128/mBio.01220-16

**Published:** 2016-09-06

**Authors:** Sebastian Felgner, Michael Frahm, Dino Kocijancic, Manfred Rohde, Denitsa Eckweiler, Agata Bielecka, Emilio Bueno, Felipe Cava, Wolf-Rainer Abraham, Roy Curtiss, Susanne Häussler, Marc Erhardt, Siegfried Weiss

**Affiliations:** a Department of Molecular Immunology, Helmholtz Centre for Infection Research, Braunschweig, Germany; b Central Facility for Microscopy, Helmholtz Centre for Infection Research, Braunschweig, Germany; c Department of Molecular Bacteriology, Helmholtz Centre for Infection Research, Braunschweig, Germany; d Department of Molecular Biology, Umeå University, Umeå, Sweden; e Department of Chemical Microbiology, Helmholtz Centre for Infection Research, Braunschweig, Germany; f Department of Infectious Diseases and Pathology, University of Florida, Gainesville, Florida, USA; g Junior Research Group Infection Biology of *Salmonella*, Helmholtz Centre for Infection Research, Braunschweig, Germany; h Institute of Immunology, Medical School Hannover, Hannover, Germany

## Abstract

Recombinant attenuated *Salmonella enterica* serovar Typhimurium strains are believed to act as powerful live vaccine carriers that are able to elicit protection against various pathogens. Auxotrophic mutations, such as a deletion of *aroA*, are commonly introduced into such bacteria for attenuation without incapacitating immunostimulation. In this study, we describe the surprising finding that deletion of *aroA* dramatically increased the virulence of attenuated *Salmonella* in mouse models. Mutant bacteria lacking *aroA* elicited increased levels of the proinflammatory cytokine tumor necrosis factor alpha (TNF-α) after systemic application*.* A detailed genetic and phenotypic characterization in combination with transcriptomic and metabolic profiling demonstrated that Δ*aroA* mutants display pleiotropic alterations in cellular physiology and lipid and amino acid metabolism, as well as increased sensitivity to penicillin, complement, and phagocytic uptake. In concert with other immunomodulating mutations, deletion of *aroA* affected flagellin phase variation and gene expression of the virulence-associated genes *arnT* and *ansB*. Finally, *ΔaroA* strains displayed significantly improved tumor therapeutic activity. These results highlight the importance of a functional shikimate pathway to control homeostatic bacterial physiology. They further highlight the great potential of *ΔaroA*-attenuated *Salmonella* for the development of vaccines and cancer therapies with important implications for host-pathogen interactions and translational medicine.

## IMPORTANCE

Recombinant attenuated bacterial vector systems based on genetically engineered *Salmonella* have been developed as highly potent vaccines. Due to the pathogenic properties of *Salmonella*, efficient attenuation is required for clinical applications. Since the hallmark study by Hoiseth and Stocker in 1981 (S. K. Hoiseth and B. A. D. Stocker, Nature 291:238–239, 1981, http://dx.doi.org/10.1038/291238a0), the auxotrophic Δ*aroA* mutation has been generally considered safe and universally used to attenuate bacterial strains. Here, we are presenting the remarkable finding that a deletion of *aroA* leads to pronounced alterations of gene expression, metabolism, and cellular physiology, which resulted in increased immunogenicity, virulence, and adjuvant potential of *Salmonella.* These results suggest that the enhanced immunogenicity of *aroA*-deficient *Salmonella* strains might be advantageous for optimizing bacterial vaccine carriers and immunotherapy. Accordingly, we demonstrate a superior performance of Δ*aroA Salmonella* in bacterium-mediated tumor therapy. In addition, the present study highlights the importance of a functional shikimate pathway to sustain bacterial physiology and metabolism.

## INTRODUCTION

Infectious diseases remain a major health problem worldwide. Despite the existence of many antimicrobial drugs and an increasing knowledge of pathogen genetics, metabolism, and host-pathogen interaction, approximately 300 million new infections and over 10 million deaths occur worldwide every year (1). Diseases like tuberculosis, malaria, or HIV infection are still major killers. Insufficient hygienic conditions but also emerging multiresistant variants of common pathogens are responsible for this. Lack of efficacious vaccines for particular infectious agents is another reason (2). As prophylactic protection by vaccination is the most appropriate and cost-effective measure against infectious disease, the development of proper vaccines and immunization strategies is one of the most challenging issues of contemporary biomedical research. In addition, newly emerging infectious agents demonstrate the need for easy-to-handle and efficacious vaccines.

*Salmonella* sp. have been considered for a live vaccine carrier for several decades (3, 4). Live bacteria have many advantages over other approaches to immunization: (i) bacteria are simple to propagate *in vitro*; (ii) bacteria can be applied orally, thus avoiding sterile needle injections and the need for specially trained personnel; and (iii) bacteria do not require cold chains as they can be transported in a lyophilized state. In addition, the complete genome sequences of several strains are known and the molecular genetics to modify those bacteria are well established.

Recombinant attenuated *Salmonella* has been shown to trigger strong cellular and humoral immune responses against pathogenic bacteria and viruses as well as cancer (5, 6). However, while the use of intrinsically pathogenic *Salmonella* strains as live vaccine carriers might be advantageous to obtain strong adjuvant activity (7, 8), the general pathogenic properties must be controlled to prevent killing the vaccinees. This demonstrates the basic dilemma of such live vaccine carriers. Attenuation and immunostimulatory capacity in the form of virulence need to be well balanced to guarantee safety and efficacy. Accordingly, it has been observed that, for *Salmonella*, a decrease in adjuvant capacity correlated with the degree of attenuation (9).

To fine-tune adjuvant capacities and attenuation, several modulating strategies have been developed. Modifications of major virulence factors, such as lipopolysaccharide (LPS) or the type III injectisome system, were employed to attenuate *Salmonella* (9, 10). In addition, to ensure persistence of immunostimulatory pathogen-associated molecular patterns (PAMPs), metabolic mutations affecting cell wall (Δ*asd*), nucleotide (Δ*purI*), or amino acid (Δ*aroA*) synthesis were used to attenuate *Salmonella* (11–13). The combination of metabolic mutations and modifications of virulence factors culminated recently in a so-called delayed lysis system, which maintained the wild-type (Wt) phenotype of *Salmonella in vitro* but became self-limiting *in vivo* (14).

A deletion of *aroA* is most commonly used as a metabolic mutation to attenuate *Salmonella* as well as other bacteria (15). AroA is part of the shikimate pathway, which directly connects glycolysis to the synthesis of aromatic amino acids (16). Thus, deletion of *aroA* renders *Salmonella* auxotrophic for aromatic amino acids, which are not freely available in the mammalian host. Consequently, *aroA*-deficient *Salmonella* strains are presumed to be highly attenuated and have been considered suitable vector systems (17).

Interestingly, a recent study showed that an interruption of the shikimate pathway in *Salmonella* not only resulted in auxotrophy but also increased sensitivity toward albumen or EDTA (18). Furthermore, an upregulation of *murA* was observed, which might explain the increased susceptibility toward albumen and EDTA, because MurA shares substrates with enzymes involved in synthesis of lipid A and the O antigen (19). Deletion of *aroA* may also influence the ubiquinone pathway that is known to influence the membrane composition (20).

In the present study, we investigated the effects of *aroA* deficiency in the context of additional immunomodulatory mutations and realized that the introduction of Δ*aroA* into our *Salmonella* strains dramatically altered their phenotype and pathogenic properties *in vitro* as well as *in vivo.* The molecular basis for these phenotypic changes was characterized by transcriptional profiling, genetic engineering, and metabolic labeling. Importantly, besides the metabolic attenuation, we observed an increased immunostimulatory capacity, and therefore, pathogenicity in the murine host was greatly enhanced in the absence of *aroA*. Thus, these strains were highly efficient in tumor therapeutic approaches, and we conclude that attenuated bacteria based on Δ*aroA* mutation might indeed prove to become optimal vector systems for vaccination and cancer therapy.

## RESULTS

### Deletion of *aroA* increases pathogenicity and immunogenicity of *Salmonella enterica* serovar Typhimurium *in vivo.*

We aimed to generate an attenuated *Salmonella* strain for use in bacterium-mediated cancer therapy. As described before, we used the highly immunogenic strain SF100 (originally χ9845), which harbors LPS with homogeneously hexa-acylated lipid A (see Table S1 in the supplemental material). For further attenuation, we introduced a deletion of *rfaG* resulting in the truncated LPS structure of strain SF103. As an additional safety feature, we deleted the frequently used gene *aroA* for metabolic attenuation (17), thereby generating strain SF104.

**FIG 1:**
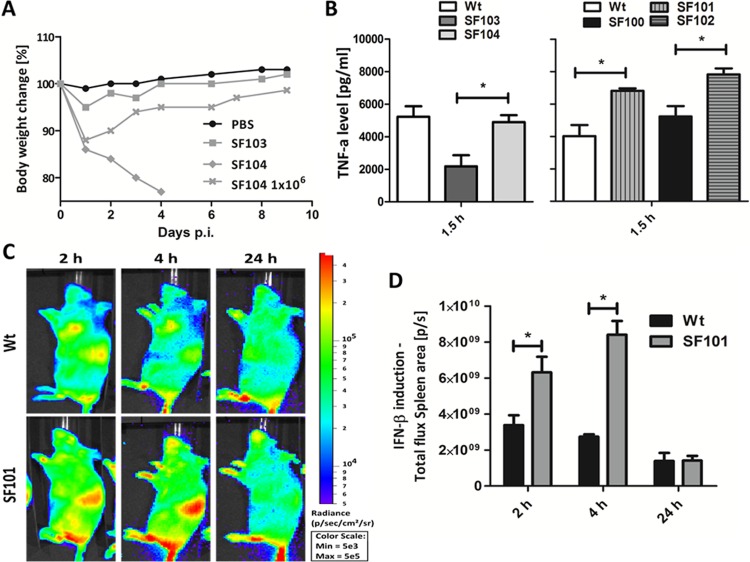
*In vivo* behavior of *aroA*-deficient *Salmonella* variants. (A) Body weight measurement as an indicator of the general health of mice infected with 5 × 10^6^ cells of SF103 (Δ*lpxR9* Δ*pagL7* Δ*pagP8* Δ*rfaG42*) or SF104 (Δ*lpxR9* Δ*pagL7* Δ*pagP8* Δ*aroA* Δ*rfaG42*) and 1 × 10^6^ cells of SF104. (B) TNF-α levels in the sera of mice, 1.5 h after infection with Wt, SF100 (Δ*lpxR9* Δ*pagL7* Δ*pagP8*), SF101 (Δ*aroA*), SF102 (Δ*lpxR9* Δ*pagL7* Δ*pagP8* Δ*aroA*), SF103, or SF104. (C and D) Determination of IFN-β induction using IFN-β reporter mice 2 h, 4 h, and 24 h postinfection. The means ± standard deviations are displayed. Results are representative for two independent experiments with 5 replicates per group. *, *P* < 0.05.

BALB/c mice were infected with SF103 and SF104, and body weight loss as a general health indicator was monitored for 2 weeks (Fig. 1). As expected, the *rfaG*-deficient strain SF103 (Δ*lpxR9* Δ*pagL7* Δ*pagP8* Δ*rfaG42*) was highly attenuated as evidenced by a minor loss of body weight. However, to our surprise, BALB/c mice succumbed to SF104 (Δ*lpxR9* Δ*pagL7* Δ*pagP8* Δ*rfaG42* Δ*aroA*) within 4 days after intravenous (i.v.) infection (Fig. 1A). This finding was in line with increased tissue burdens of SF104 compared to SF103, in particular 36 h postinfection (hpi) (see Fig. S1A and B in the supplemental material). Only a 5-fold reduction of the infection dose allowed the mice to survive, although a severe reduction of body weight was observed.

We next measured serum tumor necrosis factor alpha (TNF-α) levels as diagnostic markers for cytokine induction 1.5 h after i.v. injection of *Salmonella*. TNF-α levels in sera of mice exposed to SF104 were comparable to levels induced upon Wt infection, adding evidence for an increased immunostimulatory capacity of the *aroA* deletion strain (Fig. 1B, left panel).

To corroborate this observation, mice were infected with SF101 bearing only an *aroA* deletion and SF102 (Δ*lpxR9* Δ*pagL7* Δ*pagP8* Δ*aroA*), which harbors, besides deletion of *aroA*, homogeneous lipid A but lacks the LPS truncation of the *rfaG* mutant. Interestingly, induction of TNF-α was significantly increased in both cases compared to Wt and SF100, respectively (Fig. 1B, right panel). Similar results were obtained when the *aroA* deletion was introduced into strain ATCC 14028, demonstrating that the observed increased immunogenicity of Δ*aroA* is not restricted to one particular *Salmonella* background (see Fig. S1E in the supplemental material). As the tissue loads for the two strains were comparable during the early stage of infection, we can exclude enhanced lysis and increased release of endotoxin by the Δ*aroA* strains to explain the elevated cytokine storm (see Fig. S1A and C).

Along these lines, induction of beta interferon (IFN-β) by the bacteria was measured as an indicator for an inflammatory response of the host by employing our recently established IFN-β reporter mice (21). At 2 h and 4 h postinfection, SF101 induced higher IFN-β expression in the spleen, one of the target organs of *Salmonella*, than did the Wt (Fig. 1C and D). The same results were obtained using SF100 and SF102, respectively (see Fig. S1F and G in the supplemental material). We thus concluded that deletion of *aroA* not only renders *Salmonella* auxotrophic for aromatic amino acids but also alters its immunogenic and pathogenic properties.

### Deletion of *aroA* increases sensitivity of *Salmonella* toward membrane and periplasmic stress and decreases motility.

**FIG 2:**
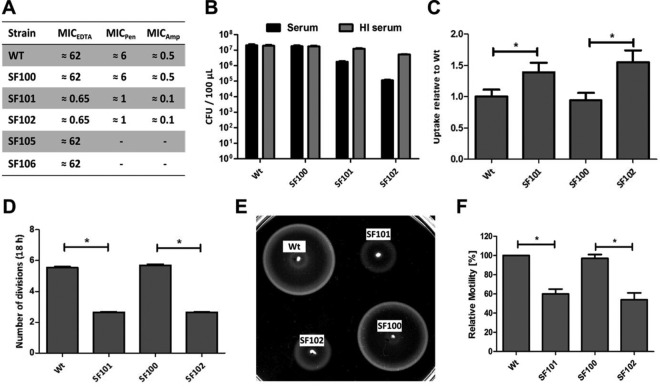
Phenotypic characterization of *aroA* mutants. (A) MIC values for EDTA (millimolar), penicillin (Pen; micrograms per milliliter), and ampicillin (Amp; micrograms per milliliter) of Wt and *aroA*-deficient strains SF101 (Δ*aroA*) and SF102 (Δ*lpxR9* Δ*pagL7* Δ*pagP8* Δ*aroA*) as well as complemented strains SF105 (Δ*aroA p-aroA*) and SF106 (Δ*lpxR9* Δ*pagL7* Δ*pagP8* Δ*aroA p-aroA*). (B) *In vitro* sensitivity toward human complement. Bacteria (2 × 10^7^) were treated with either untreated or heat-inactivated (HI) human serum for 30 min at 37°C. The lysis effect was determined by plating. (C) Phagocytic uptake of Wt and Δ*aroA* variants. J774 cells were infected with an MOI of 1, and uptake was determined relative to Wt after 1 h. (D) Intracellular replication of Wt and Δ*aroA* variants in J774 cells. Cells were allowed to engulf the bacteria as in the experiment depicted in panel C. Infected cells were incubated for 18 h, and the remaining numbers of bacteria were determined by plating. (E and F) Motility was assessed on semisolid agar. Means ± standard deviations are displayed. Results are representative for two independent experiments with 5 biological replicates per group. *, *P* < 0.05.

We hypothesized that alterations of the cell envelope might explain the increase in pathogenicity and immunogenicity of the Δ*aroA* variants. Hence, we tested the sensitivity of our strains against membrane-active reagents like EDTA. Δ*aroA* mutants of *S*. Typhimurium strain LT2 had been shown before to be 10 times more sensitive to EDTA than Wt (18). In accordance, SF101 and SF102 were 100 times more sensitive towards EDTA than was the Wt strain UK-1 or SF100. Similarly, strains lacking *aroA* were more sensitive to penicillin and ampicillin. Complementation of the *aroA* deletion by plasmid-carried *aroA* (SF105 and SF106) rescued resistance of *ΔaroA* mutants to EDTA (Fig. 2A). Of note, Δ*aroC* (SF137) and Δ*aroD* (SF138) mutants exhibit similar EDTA sensitivities, indicating that the lack of this metabolic pathway leads to the phenotype and is not specific for Δ*aroA* (see Fig. S2A in the supplemental material).

Similarly, Δ*aroA* mutants were tested for resistance against effector mechanisms of the innate immune system. When exposed to complement of human sera, bacteria lacking *aroA* (SF101 and SF102) were significantly more sensitive than Wt and SF100 (Fig. 2B). Furthermore, Wt and SF100 were more resistant to phagocytic uptake by the macrophage-like cell line J774 (Fig. 2C). On the other hand, Wt and SF100 were more capable of surviving intracellularly than SF101 and SF102 lacking *aroA* (Fig. 2D). We thus concluded that *aroA* mutant strains exhibit severe alterations of the cell envelope.

Previously, we had shown that cell envelope integrity and alterations of the LPS influenced motility of *Salmonella* (9, 22). Thus, we tested the motility of the *aroA*-deficient strains. As displayed in Fig. 2E and F, SF101 and SF102 harboring *ΔaroA* were significantly less motile than the corresponding parental strains. The same phenotype was observed for Δ*aroC* (SF137) and Δ*aroD* (SF138) strains (see Fig. S2B in the supplemental material).

In an attempt to visualize potential alterations of the outer membrane of *ΔaroA* mutants, we employed electron microscopy of negatively stained bacteria (see Fig. S3A in the supplemental material). No differences were observed between Wt and SF101. Flagella were visible for both strains, although motility was impaired for SF101.

Taken together, these *in vitro* results demonstrate that the deletion of genes of the shikimate pathway, like *aroA*, *aroC*, or *aroD*, exerts a pleiotropic effect on the membrane status of *Salmonella.* Complementation of *ΔaroA* restored the original wild-type phenotype concerning pathogenicity, intracellular survival, and complement resistance, indicating that the alterations were indeed caused by the absence of *aroA*, i.e., a functional shikimate pathway (see Fig. S3B to E in the supplemental material).

### Putative defects in ubiquinone synthesis can only partially explain the Δ*aroA* phenotype.

Recently, it has been shown that ubiquinone deficiency affects cell envelope stability (20). Ubiquinones derive from chorismate. Hence, the observed phenotype of Δ*aroA* mutants may be caused by ubiquinone deregulation. To address this question, the genes *ubiG* (SF140) and *ubiA* (SF141) were deleted in *Salmonella*. Interestingly, these mutants were more sensitive to EDTA than the Δ*aroA* mutant SF101 (see Fig. S4A in the supplemental material). However, the *in vitro* growth of SF140 and SF141 in LB medium was significantly impaired (see Fig. S4B). This argued that the ubiquinone dysregulation might be only partially responsible for the *in vitro* Δ*aroA* phenotype of SF101. Furthermore, genes of the ubiquinone pathway were not differentially regulated (see below).

To evaluate whether deficiency in the ubiquinone pathway would have an impact on the immunogenicity of *Salmonella in vivo*, TNF-α levels were measured in serum of mice 1.5 h after i.v. infection by SF140 and SF141. TNF-α levels induced by these bacteria were significantly lower than those induced by Wt and SF101 (see Fig. S4C in the supplemental material). This correlated with negligible body weight loss upon infection, indicating that mutants deficient in ubiquinone synthesis are not viable *in vivo*. Altogether, the *in vivo* phenotype of *aroA*-deficient mutants does not resemble that of ubiquinone mutants, although the ubiquinone synthesis pathway is situated downstream of chorismate. However, it appears possible that disturbances in ubiquinone synthesis may contribute to the observed alterations of membrane integrity.

### Differential turnover of fatty and amino acids in Wt and *ΔaroA* variants.

Fatty acids (FAs) are an essential part of bacterial membranes. Variations in their composition could be responsible for the observed phenotype. Therefore, we analyzed the FA composition of *ΔaroA* mutants in steady state by high-resolution gas chromatography. The fatty acid methyl ester (FAME) profiles were in general comparable between the Δ*aroA* mutants and their corresponding *aroA^+^* parental strains. Only a significant decrease in the amount of heptadecenoic acid (c17:1ω6) was observed for the Δ*aroA* mutants SF101 and SF102 (see Fig. S5 in the supplemental material). Its contribution to the observed phenotype remains unclear.

**FIG 3:**
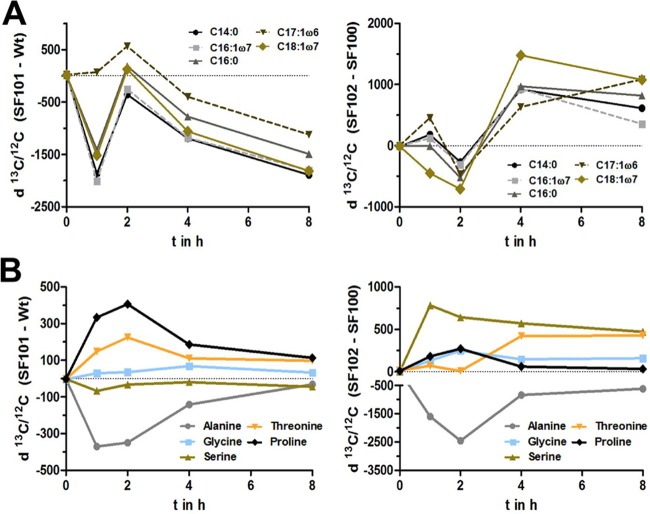
Differential turnover of fatty and amino acids in Wt and Δ*aroA* variants. (A) ^13^C incorporation into fatty acids. *Salmonella* bacteria were fed with labeled glucose, and the ^13^C/^12^C ratios of the fatty acids were measured. The values of the parental strain were subtracted from those of the *aroA* variants. Positive values indicate that the metabolic turnover from ^13^C-labeled glucose to fatty acid was significantly higher than that for the parental strain. (B) ^13^C incorporation into amino acids. Left, difference of SF101 (Δ*aroA*) from Wt. Right, difference of SF102 (Δ*lpxR9* Δ*pagL7* Δ*pagP8* Δ*aroA*) from SF100 (Δ*lpxR9* Δ*pagL7* Δ*pagP8*). The means with standard deviations are displayed. Results are representative for two independent experiments with 3 biological replicates per group.

We next investigated the turnover of FAs by cultivating the bacteria in media containing ^13^C-labeled glucose. In contrast to the steady-state profile, the metabolic turnover of [^13^C]glucose was significantly changed for *aroA* mutants (Fig. 3A). The incorporation of ^13^C into FAs was slower and delayed, demonstrating that deletion of *aroA* significantly alters the kinetics of FA synthesis. Increased incorporation was observed only for fatty acid c17:1ω6 (see Fig. S5 in the supplemental material). Note that the lipid A modification of strain SF100 also affected FA synthesis, although the *aroA* deletion exhibited a dominant effect and resulted in delayed incorporation (see Fig. S5).

The cellular fraction of FA was analyzed in detail by separation into phospholipids, glycolipids, and neutral lipids. In particular, the turnover of phospholipids and neutral lipids was significantly delayed in *ΔaroA* variants (data not shown). We concluded that the altered synthesis of phospholipids as major components of the cell envelope could explain the increased sensitivity to membrane-acting compounds (Fig. 2A to C).

Due to the sensitivity of Δ*aroA* to penicillin, the peptidoglycan composition of the cell wall was analyzed using ultraperformance liquid chromatography (UPLC) chromatography. However, no significant differences were revealed (see Fig. S5E in the supplemental material). Thus, we concluded that the outer membrane but not the cell wall composition is affected in the Δ*aroA* strains.

We next investigated the turnover of representative amino acids using incorporation of [^13^C]glucose (Fig. 3; see also Fig. S5 in the supplemental material). The synthesis of the majority of analyzed amino acids was not significantly altered. However, the synthesis of alanine was negatively affected in the *aroA* variants as judged by significantly reduced incorporation of [^13^C]glucose (Fig. 3B). In contrast, synthesis of proline, threonine, or glycine was enhanced in the Δ*aroA* mutants. In addition, the incorporation into serine was altered only for strains SF100 and SF102, which express a modified lipid A (Fig. 3B, right panel).

In summary, the interruption of the shikimate pathway by deletion of *aroA* has a global effect on the metabolism of *Salmonella* as exemplified here for fatty acids and amino acid metabolisms, indicating a severe metabolic stress.

### Δ*aroA* modulates the genetic profile of *S*. Typhimurium.

**FIG 4:**
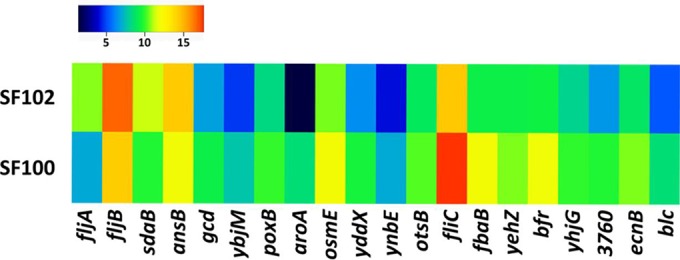
*In vitro* transcriptome analysis of SF100 (Δ*lpxR9* Δ*pagL7* Δ*pagP8*) and SF102 (Δ*lpxR9* Δ*pagL7* Δ*pagP8* Δ*aroA*). Expression profile of the most prominent differentially regulated genes in the *aroA*-deficient mutant SF102 in comparison to its parental strain SF100. Normalized reads for particular genes are shown.

In a complementary approach to the physiological investigations and metabolic profiling, we performed *in vitro* transcriptome analyses of the *aroA* mutants. Transcriptome data of strain SF101 revealed an in-frame deletion of the Mu phage-like region (STMUK_1978 to STMUK_2030) and were not analyzed further. We focused on transcriptome analysis of strains SF100 and SF102, which represent the basis of our therapeutic attempts. In addition, the observed phenotypes of strains SF101 and SF102 were very similar, as described above. In total, 104 genes (22 upregulated, 82 downregulated) were differentially regulated in the Δ*aroA* mutant strain SF102 compared to the parental *aroA^+^* strain SF100 (Fig. 4). The differentially regulated genes could be classified into four major functional pathways: (i) metabolism of sugars, amino acids, and lipoproteins; (ii) osmoregulation; (iii) virulence; and (iv) flagellar biosynthesis.

Metabolic pathways responsible for the synthesis of mannose (*manXYZ*) and lipoproteins (e.g., *ecnB*, *blc*, *ynbE*, etc.) were downregulated in the absence of *aroA*, while the glycerophospholipid metabolism (*glpQT*) was significantly upregulated (see Table S2 in the supplemental material, GSE74433). These molecules are part of the cell envelope and extracellular structures, and we concluded that the altered expression might be relevant for the increased susceptibility to membrane-active reagents or macrophages. Furthermore, glycolysis was negatively regulated, suggesting that excessive pyruvate is available in mutants deficient for the shikimate pathway. Consistently, intracellular pyruvate levels were increased in the *aro* mutants (see Fig. S6A).

Second, we observed significantly decreased expression of the genes *otsAB*, *osmE*, and *yehVWXYZ* involved in osmoregulation. This suggested that altered sugar production might be sufficient to compensate for the increased pyruvate levels. Alternatively, such sugars may lead to an osmotic imbalance that causes physiological stress for the bacteria. This hypothesis was supported by differential regulation of many transporter systems (see Table S2 in the supplemental material). In addition, the concentration of trehalose-6-phosphate, a sugar molecule that regulates osmotic pressure, was significantly lower in the Δ*aroA* mutants (see Fig. S6B). Finally, as expected from the altered turnover of FAs and amino acids, the expression levels of genes involved in the respective metabolism/synthesis pathways were altered.

Interestingly, the transcriptome analysis revealed differentially regulated virulence factors, which may contribute to the increased pathogenic/immunogenic phenotype of the *aroA* mutants. The gene *ansB* was significantly upregulated in SF102. AnsB is known to interfere with T-cell responses (23), and overproduction of AnsB could explain the increased pathogenicity of Δ*aroA* in the *rfaG*-deficient background *in vivo* (Fig. 1A).

### Δ*aroA* is biased to FljB phase 2 flagellin orientation in a hexa-acylated lipid A environment.

**FIG 5:**
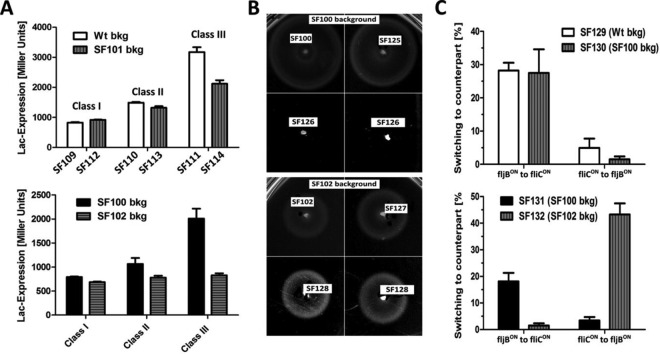
Flagellar phenotype of Δ*aroA* strains. (A) β-Galactosidase assay to measure activity of fusion of *mudJ* with genes of the different flagellar gene classes. Class I (*flhDC5213*-*mudJ*), class II (*fliL5100-mudJ*), and class III (*fljB5001*::*mudJ* Δ*hin5718*::FRT) constructs were on a Wt (SF109, SF110, and SF111), SF101 (SF112, SF113, and SF114), SF100 (SF115, SF116, and SF117), or SF102 (SF118, SF119, and SF120) background (bkg), respectively. (B) Swimming assay for *fliC* deletion mutants SF126 (Δ*lpxR9* Δ*pagL7* Δ*pagP8* Δ*fliC*::FCF) and SF128 (Δ*lpxR9* Δ*pagL7* Δ*pagP8* Δ*aroA ΔfliC*::FCF) compared to parental strains SF102 and SF100, respectively. SF127 and SF125 served as positive controls for constitutively expressed *fljB*. (C) Preferential phase switching from *fliC* (Lac^+^) to *fljB* (Lac^−^) or vice versa. Lac^+^ or Lac^−^ colonies were cultured and plated after 4 h on TTC (2,3,5-triphenyltetrazolium chloride) plates. The switching was assayed by counting Lac^+^ and Lac^−^ colonies developed from either *fliC* or *fljB* strains. The means with standard deviations are displayed. Results are representative for two independent experiments with 5 biological replicates per group.

Since we observed a motility defect of *aroA*-deficient strains, we analyzed the flagellar phenotype in detail. The flagellar regulon is organized in a transcriptional hierarchy of three promoter classes (class I, class II, and class III). We used transcriptional *lacZ* fusion to representative genes that are under the control of class I, class II, or class III promoters. Expression levels from class I (*flhC*) and class II (*fliL*) promoters were similar in the Wt and *aroA* mutant, whereas a significant downregulation of class III gene expression was observed (*fljB*) in both SF101 and SF102 (Fig. 5A). The lower expression of the *fljB* phase 2 flagellin correlated with the reduced motility (Fig. 2F).

Interestingly, the flagellin locus orientation was found to be affected by *ΔaroA* but only in the context of hexa-acylated lipid A of strain SF102 (Δ*lpxR9* Δ*pagL7* Δ*pagP8* Δ*aroA*). This indicates a joined effect of the *aroA* deletion and the lipid A modification on the transcriptional regulation of flagella. Of note, transcriptome analysis of Wt and the corresponding hexa-acylated lipid A mutant SF100 (Δ*lpxR9* Δ*pagL7* Δ*pagP8*) did not reveal any regulatory differences. However, the flagellin phase 2 gene *fljB* and the flagellin phase 1 repressor gene *fljA* were significantly upregulated in the isogenic *aroA* deletion strain SF102. In addition, the expression level of the DNA invertase *hin* was reduced (see Table S2 in the supplemental material). These data indicated that flagellar biosynthesis was altered in SF102. Components of the flagellum are known to affect the pathogenicity and immunogenicity of *Salmonella.* As this might be important for our therapeutic approach, we consequently investigated flagellar biosynthesis and composition in detail.

Our transcriptome data showed upregulation of *fljB* in the *aroA* mutant strain SF102, whereas *fliC* expression was reduced in comparison to the parental strain SF100 (see Table S2 in the supplemental material). This finding was supported by motility analyses of mutants lacking *fliC* (SF126 and SF128) as shown in Fig. 5B. Strain SF100 expresses preferentially flagellin phase 1 (FliC), since the isogenic *fliC* mutant SF126 (Δ*lpxR9* Δ*pagL7* Δ*pagP8* Δ*fliC*::FCF) was nonmotile. In accordance, the deletion of *fliC* in Wt or SF101 resulted also in a nonmotile phenotype (data not shown). In contrast, the deletion of *fliC* in the *aroA* deletion strain SF128 (Δ*lpxR9* Δ*pagL7* Δ*pagP8* Δ*aroA* Δ*fliC*::FCF) did not change the motility phenotype compared to the parental strain SF102 (Fig. 5B). Together with the transcriptome data, these results indicated that the Δ*aroA* strain SF102 preferentially expressed flagellin phase 2 (FljB).

To corroborate these findings, we determined the switching frequency from flagellin phase 1 (FliC) to flagellin phase 2 (FljB) and vice versa as described before (24). In the absence of *aroA*, almost 50% of the FliC-positive colonies of SF102 displayed a bias to switch to the *fljB*-ON orientation, while less than 2% switched from *fljB*-ON to *fliC*-ON (Fig. 5C). In contrast, the three other strains preferably switched to *fliC*-ON, which is consistent with previous reports on flagellin phase-switching frequencies (24).

Taken together, the expression of flagellar genes is differentially regulated in the Δ*aroA* mutant strains SF102 and SF101. In addition, SF102 displayed a bias for flagellin phase 2 (*fljB*-ON) orientation. Thus, the switch to FljB is caused by a synergism between *aroA* and the lipid A. Both gene deletions are apparently involved in the modification of the cell envelope (Fig. 3; see also Fig. S5 in the supplemental material).

### *In vivo* transcriptome analysis revealed *arnT* as a possible contributor to the cytokine storm.

**FIG 6:**
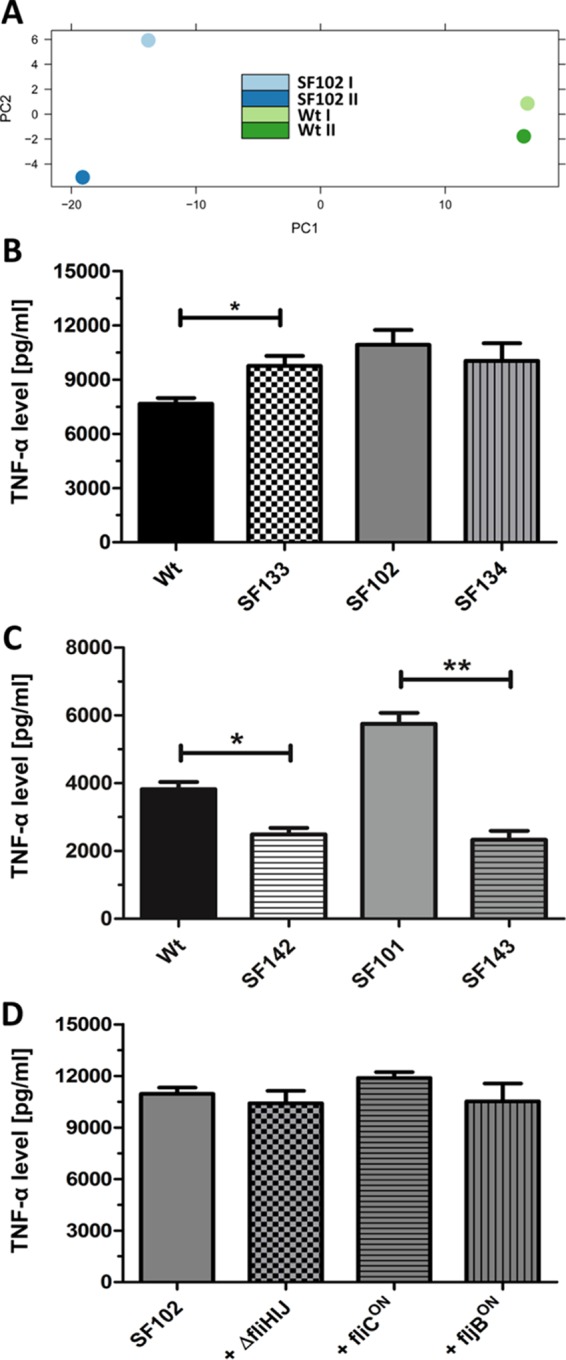
*In vivo* transcriptome analysis of Wt and SF102 (Δ*lpxR9* Δ*pagL7* Δ*pagP8* Δ*aroA*). (A) Principal component analysis. (B) TNF-α levels in the sera of mice infected with Wt, SF102 (Δ*lpxR9* Δ*pagL7* Δ*pagP8* Δ*aroA*), SF133 (Δ*arnT*::FKF), or SF134 (Δ*lpxR9* Δ*pagL7* Δ*pagP8* Δ*aroA* Δ*arnT*::FKF) 1.5 h postinfection. (C) TNF-α levels in the sera of mice infected with Wt, SF101 (Δ*aroA*), SF142 (Δ*ansB*::FKF), or SF143 (Δ*aroA* Δ*ansB*::FKF). (D) Effect of flagellar phenotype on TNF-α induction. The means with standard deviations are displayed. Results are representative for two independent experiments with 5 replicates per group. *, *P* < 0.05; **, *P* < 0.01.

The *in vitro* transcriptome analysis revealed major changes in the metabolism of bacteria that lack *aroA*. Therefore, we performed transcriptome profiling from tumor-residing *Salmonella ex vivo.* Due to the identical transcriptomes of Wt and SF100 *in vitro*, we conducted the *ex vivo* transcriptome comparison for Wt and SF102 (Fig. 6A). To normalize conditions, RNA was extracted from tumors that were colonized equally with *Salmonella* (data not shown). Expression of 530 genes was differentially altered. Importantly, genes that were differentially regulated *in vitro* showed the same expression pattern in tumor-colonizing bacteria (see Fig. S7 in the supplemental material). In addition, various virulence factors (e.g., *inv*, *sop*, and *ssa*) were downregulated in SF102 *in vivo* (see Table S3), indicating loss of certain virulence properties *in vivo*. These data show that the pleiotropic impact of Δ*aroA* on gene expression *in vitro* is replicated in *in vivo* environments.

Of note, the gene *arnT* was significantly downregulated *in vivo.* This indicated changes in the lipid A structure of the *aroA* deletion mutant (see Table S3 in the supplemental material). ArnT masks the 4′-phosphate group of lipid A, and accordingly, lipid A recognition by the TLR4-MD2 receptor complex is minimized (25). A reduced *arnT* expression in the *aroA* mutant SF102 might result in enhanced triggering of TLR4 and would thus explain the increased immunogenicity (Fig. 1B and C).

To validate the impact of *arnT*, deletion strains SF133 (Δ*arnT*::FKF) and SF134 (Δ*lpxR9* Δ*pagL7* Δ*pagP8* Δ*aroA* Δ*arnT*::FKF) were tested in mice and the serum concentration of TNF-α was measured as a marker for the elicited cytokine storm. As expected, *arnT* deficiency increased the immunostimulatory capacity of SF133 in comparison to Wt (Fig. 6B). No additive effect of the *arnT* deletion was observed for TNF-α levels on the *aroA* mutant background of strain SF102 (Fig. 6B). This suggests that TNF-α induction in the *aroA* mutant was already at its maximum due to downregulation of *arnT*.

*In vitro*, *ansB* was found to be upregulated in the *aroA* mutants. As *ansB* is already known to affect adaptive immunity, we wondered whether it could also influence the initial innate immune response. Thus, the gene for *ansB* was deleted in Wt and SF101, resulting in strains SF142 (Δ*ansB*::FKF) and SF143 (Δ*aroA* Δ*ansB*::FKF), respectively. Interestingly, lack of *ansB* resulted in a significant reduction of TNF-α levels *in vivo* (Fig. 6C). Therefore, the upregulation of *ansB* may add to the increased immunogenicity of the *aro* mutants.

Components of the flagellum have previously been shown to be immunogenic. Therefore, flagellar mutant Δ*fliHIJ* (no filament) and flagellin phase-locked mutant *fliC*-ON or *fljB*-ON were generated on the *aroA* mutant background of strain SF102. As shown in Fig. 6C, no significant differences in TNF-α levels between the mutants and the parental *aroA* mutant strain were observed, indicating a low impact of flagella on immunogenicity under these conditions.

### Δ*aroA* significantly contributes to a successful cancer therapy using attenuated *Salmonella.*

**FIG 7:**
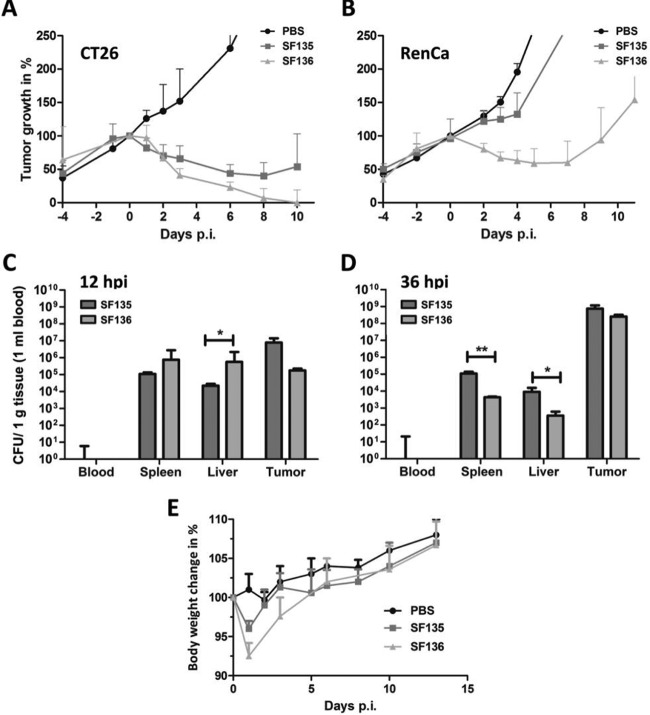
Therapeutic benefit of Δ*aroA* in bacterium-mediated tumor therapy. Immunogenic CT26 (A)- and aggressive RenCa (B)-bearing mice were infected i.v. with 5 × 10^6^ SF135 (Δ*rfaG*) or SF136 (Δ*aroA* Δ*rfaG*) bacteria. The tumor volume was monitored, and the medians with ranges are displayed. (C and D) Blood, spleen, liver, and tumor bacterial burdens of CT26-bearing mice were determined by plating serial dilutions of tissue homogenates. CFU counts of SF135 and SF136 at 12 hpi (C) and 36 hpi (D). (E) Body weight measurement as indicator for general health status upon infection with LPS variants. Results are representative for two independent experiments with 5 replicates per group. *, *P* < 0.05; **, *P* < 0.01.

As demonstrated above, deletion of *aroA* increased the immunostimulatory properties of *Salmonella*. Therefore, a boost of the adjuvant effects during therapeutic approaches might be produced. To address this question, we modified previously described LPS mutants (9) for bacterium-mediated tumor therapy by deleting *aroA*. As shown before, the *rfaG* mutant SF135 is able to target CT26 tumors and retard their growth. However, no tumor clearing was observed (Fig. 7). In contrast, an Δ*aroA* Δ*rfaG* double mutant (SF136) was able to target CT26 tumors and completely clear the tumors (Fig. 7A). Importantly, aggressive RenCa tumors were also targeted by both strains, and the growth of RenCa was significantly delayed upon infection with the *aroA*-deficient strain SF136 (Fig. 7B). In line with results described above, the deletion of *aroA* increased the pathogenicity of SF136 during early stages of infection. The bacterial burden in spleen and liver was enhanced at 12 hpi (Fig. 7C). This was also reflected in the increased initial body weight drop of the mice (Fig. 7E). However, at later stages the attenuating characteristics of Δ*rfaG* and Δ*aroA* were dominating (Fig. 7D). Hence, these experiments show that deletion of *aroA* contributes significantly to the therapeutic power of *Salmonella* in bacterium-mediated tumor therapy.

## DISCUSSION

*Salmonella* Typhimurium is exploited as a versatile vehicle for vaccination as well as therapeutic purposes. It exerts strong immunogenicity due to its pathogenic nature, i.e., it expresses virulence factors that alert the immune system but might also subvert immune effector mechanisms. Thus, strong safety measures need to be applied to allow the use of these bacteria in experimental as well as clinical studies. Among many possibilities, the introduction of an auxotrophic mutation by deleting the gene *aroA* has commonly been used for metabolic attenuation. AroA is involved in the synthesis of aromatic amino acids, which are not freely available in the mammalian host. Nevertheless, such bacteria can survive to a certain extent in the host and might exert fatal virulence in immunocompromised individuals (26). Surprisingly, a highly attenuated Δ*rfaG* mutant became lethal in mice when combined with a deletion of *aroA*.

**FIG 8:**
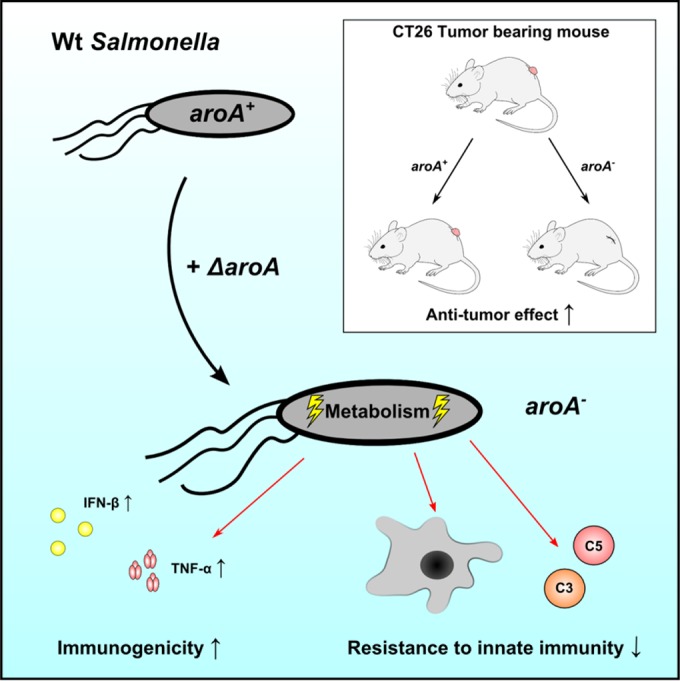
Graphic summary showing the effects of Δ*aroA* in *Salmonella*. The deletion of *aroA* acts globally on *Salmonella*, leading to increased immunogenicity, increased susceptibility toward components of the innate immune system, and increased therapeutic efficacy in bacterium-mediated tumor therapy.

Here, we investigated this unexpected effect by analyzing the *in vitro* and *in vivo* transcriptome, metabolism, and physiology of *aroA*-deficient *Salmonella*. We show that the absence of *aroA* not only metabolically attenuates the microorganisms but also exerts wide-ranging pleiotropic effects on bacterial physiology, virulence, and immunogenicity (Fig. 8).

AroA is part of the shikimate pathway. The lack of AroA interrupts the pathway that connects glycolysis to aromatic amino acid synthesis. This interruption leads to an accumulation of intracellular pyruvate by negative feedback loops in the bacteria (27) that might cause osmotic stress and could elicit all the observed alterations in turn (see Fig. S6A in the supplemental material). In agreement with this interpretation, many synthesis pathways that are differentially affected by Δ*aroA* are starting from pyruvate and may compensate for this effect. Furthermore, the downregulation of proteins upstream of pyruvate in glycolysis (e.g., Gcd, FbaB, and TalA) further supports cytoplasmic accumulation of pyruvate. As the pyruvate accumulation was observed for other *aro* deletions such as *aroC* or *aroD*, a general effect of the shikimate pathway on the physiology of *Salmonella* becomes apparent (see Fig. S6A in the supplemental material). The metabolic blockage may also alter the intracellular redox potential NADH/NAD^+^, which is known to affect cellular processes in turn (28).

The increased turnover of the amino acids serine and glycine correlates with elevated pyruvate concentrations in *aroA* deletion mutants, as they derive from 3-phosphoglycerate. We conclude that the increased turnover of these amino acids indicates a higher turnover of the tricarboxylic acid cycle. However, we expected alanine synthesis to be upregulated in *aroA* mutants in order to remove intracellular pyruvate, but the opposite was the case (Fig. 3B). Thus, we hypothesize that the alanine synthesis pathway is negatively affected by the general stress conditions in the absence of *aroA*.

The production of sugars that act as osmolytes is altered in the Δ*aroA* mutant. Such sugars may lead to further osmotic imbalance and osmostress. The excessive sugar synthesis may be an attempt by the cell to balance osmoregulation. This could also be the explanation for the significant downregulation of genes like *otsAB* and *yehVWXYZ* that are involved in osmoregulation. In support, trehalose, a regulator of osmotic pressure under normal conditions, was found in lower concentrations in *aroA* mutants. Another potential indicator for physiological stress was defects in synthesis of neutral lipids, which function as important energy storage (29).

The metabolism of phospholipids and especially glycerophospholipids was also delayed in the *aroA* mutant. Glycerophospholipids are a major component of the outer membrane and therefore crucial for membrane integrity (30). Next to glycerophospholipids, we investigated the role of ubiquinones that can affect envelope stability if their synthesis is impaired (20). Indeed, abrogation of ubiquinone synthesis increased the sensitivity to EDTA. However, the general phenotype of such variants did not resemble that of *aroA*-, *aroC*-, or *aroD*-deficient mutants. Thus, *aro*-deficient mutants are at least partially able to complement deficiencies in the ubiquinone pathway.

Altogether, the altered glycerophospholipids and putative disturbances of ubiquinone synthesis could interfere with the stability of the outer membrane and therefore provide an explanation for the sensitivity to albumen or EDTA observed by us and Sebkova et al. (18). Furthermore, it might explain the increased sensitivity to ampicillin and penicillin and the reduced intracellular survival after phagocytosis. The latter phenotype might also be influenced by the downregulation of bacterioferritin that has been reported to protect bacteria from toxic hydroxyl radicals and reactive oxygen species (ROS) in phagocytes (31).

Along the line of cell envelope changes, mannose synthesis was found to be downregulated in *aroA* deletion mutants. As the O antigen of LPS is composed of hexose sugars, lack of mannose might result in a modified primary structure of the O antigen. This could influence the recognition by macrophages and complement system, as was observed before (32).

The mutant SF101 that lacks only *aroA* exhibits the same flagellation state and bias toward the *fliC*-ON orientation as Wt *Salmonella*. Likewise, hexa-acylated lipid A alone does not result in a bias toward the *fljB*-ON orientation or affect flagellar gene expression. However, flagellar biosynthesis was altered in Δ*aroA* strains in conjunction with an optimized lipid A structure (Δ*lpxR9* Δ*pagL7* Δ*pagP8*). Therefore, the *aroA* deletion does contribute to the switch to *fljB*-ON orientation only in the double mutant with hexa-acylated lipid A and *ΔaroA*. This suggests that the *ΔaroA* phenotype becomes apparent only if amplified by hexa-acylation of lipid A. Additional experiments will be required to reveal the potential cross talk between these two modifications.

The bias toward the flagellin phase 2 (*fljB*-ON) orientation in the Δ*aroA* mutant SF102 does not explain the lack of motility. Mutants locked in either *fliC*-ON or *fljB*-ON orientation do not display motility defects (33). However, in addition, expression of class III flagellar genes (e.g., *fliC* and *fljB*) was significantly decreased. Thus, the reduced expression of flagellar genes might explain the decreased motility of the *aroA*-deficient strain.

The abovementioned alterations do not explain the increased immunogenicity and pathogenicity of the Δ*aroA* strains especially since genes connected to the type III injectisome apparatus were downregulated. The transcriptome analysis revealed *ansB* as a potential candidate gene responsible for the increased immunogenicity. *ansB* was upregulated in *ΔaroA* strains. The gene *ansB* encodes an l-asparaginase II, which catalyzes the hydrolysis of l-asparagine to aspartic acid and ammonia and has been shown to suppress T-cell-mediated immune reactions like blastogenesis, proliferation, and cytokine production (23). Furthermore, a deletion of *ansB* resulted in a reduced TNF-α induction (Fig. 6C). Therefore, the increased activity of AsnB in Δ*aroA* mutants could explain the enhanced pathogenicity and immunogenicity (Fig. 1A). In this context, recognition of flagellin by TLR5 might also play a role *in vivo*. In a previous study, it has been demonstrated that *Salmonella* expressing flagellin phase 2 (FljB) exhibited an increased adjuvant effect and boosted FljB-specific IgG responses (34). We found that the bias toward the *fljB*-ON orientation in *aroA* mutants did not contribute significantly to the increased TNF-α and IFN-β levels. However, the switch in flagellin phase might contribute to immunogenicity in infection systems other than the murine model employed here.

In general, the lipid A molecule as part of the LPS is known to play a major role in septicemia (35). Importantly, we found that the gene *arnT* was significantly downregulated in the Δ*aroA* mutants. It encodes a 4-amino-4-deoxy-l-arabinose transferase that masks the lipid A molecule *in vivo* in order to avoid recognition by TLR4 (36). While a hexa-acylated, diphosphorylated lipid A is highly immunostimulatory, tetra-acylated lipid A with masked phosphate groups acts antagonistically (25). The *aroA*-deficient *Salmonella* mutant lacks the ability to mask the 4′-phosphate group of lipid A due to the downregulation of *arnT*. In accordance, cytokine production is increased in *aroA* mutant strains (Fig. 6). Similarly, a deletion mutant of *arnT* added additional immunogenicity to the strains. However, when *arnT* was deleted in the hexa-acylated lipid A strain background SF102, no increase in pathogenicity was observed. This suggested that SF102 already exhibited the same phenotype as the Δ*arnT* mutant. In summary, we conclude that differential regulation of *arnT* and *ansB* mediates the increased immunogenicity of the Δ*aroA* mutation *in vivo*.

Finally, we hypothesized that the increased *in vivo* pathogenicity/immunogenicity might increase the therapeutic potency of the *aroA*-deficient *Salmonella* when employed in bacterium-mediated cancer therapy. Importantly, when *aroA* was deleted in the highly attenuated Δ*rfaG* mutant (SF135), we observed a significantly boosted antitumor effect. This became most apparent when the bacteria were tested in the RenCa tumor model, which usually exhibits only limited susceptibility to bacterial therapy. We conclude that tumor clearance or growth retardation might benefit from the increased induction of TNF-α by *aroA* mutants. The increased potency of these Δ*aroA* mutants is also reflected in initially higher bacterial burdens that might further stimulate the immune system.

Taken together, this study demonstrated that the commonly used deletion Δ*aroA* exerts global effects on gene expression, metabolism, and physiology of *Salmonella.* The absence of *aroA* not only renders *Salmonella* auxotrophic for aromatic amino acids but also improves its immunogenicity and adjuvant power while decreasing virulence mediated by its type III injectisome system at the same time. Therefore, we propose that the use of *aroA* deletion mutants or alternative mutants of the shikimate pathway in combination with other attenuating modifications might produce a highly optimized *Salmonella* strain for vaccination and bacterium-mediated cancer therapy.

## MATERIALS AND METHODS

### Ethics statement.

All animal experiments were performed according to the guidelines of the German Law for Animal Protection and with the permission of the local ethics committee and the local authority LAVES (Niedersächsisches Landesamt für Verbraucherschutz und Lebensmittelsicherheit) under permission no. 33.9-42502-04-12/0713.

### Bacterial strains.

Bacterial strains and plasmids are shown in Table S1 in the supplemental material. Bacteria were grown in LB or 1% (wt/vol) galactose minimal medium at 37°C. Suicide vector pYA3600 was used for *aroA* deletion in χ3761 (UK-1 Wt) and SF100 (Δ*lpxR9* Δ*pagL7* Δ*pagP8*) as described previously (37). Deletion was confirmed by PCR. P22 bacteriophage transduction was used for targeted gene deletions (38). For complementation studies, the vector pTrc99A (*P_lac_* ColE1 *ori* Amp^r^) was used and induced with 1 mM isopropyl-β-d-thiogalactopyranoside (IPTG) (39).

### Preparation of inoculum.

*Salmonella* strains were grown overnight and subcultured to mid-log phase in LB medium at 37°C. The bacteria were washed twice and adjusted to the desired optical density at 600 nm (OD_600_) (e.g., 0.055 equals 5 × 10^7^ *Salmonella* bacteria/ml) in pyrogen-free phosphate-buffered saline (PBS).

### Motility assay.

The motility of mutant strains was assayed on semisolid swimming plates containing 0.3% (wt/vol) agar and quantified by measuring the swarm diameter after a 4-h incubation at 37°C.

### Flagellar expression.

To quantify flagellar gene expression, transcriptional *lacZ* fusions to *flhDC* (class I), *fliL* (class II), and *fljB* or *fliC* (class III) were used, and *lacZ* activity was measured as described previously (24, 40).

### Electron microscopy.

Overnight cultures were fixed in 2% glutaraldehyde and negatively stained with 2% uranyl acetate. Samples were examined in a Zeiss 910 transmission electron microscope (TEM) at 80 kV with calibrated magnifications. Images were recorded with a slow-scan charge-coupled device (CCD) camera (ProScan; 1,024 by 1,024) and ITEM software (Olympus Soft Imaging Solutions).

### Trehalose measurement.

Strains were cultured overnight in 10 ml LB. Bacteria were centrifuged, washed, and resuspended in 500 µl double-distilled water (ddH_2_O). The lysate was prepared by treating the bacteria for 30 min at 95°C. The trehalose assay kit (Megazyme) was used to measure intracellular trehalose concentrations in the supernatant as described by the manufacturer.

### Pyruvate measurement.

Bacteria were cultured in 5 ml LB overnight. Bacteria (2 ml) were washed and resuspended in 300 µl ddH_2_O. The lysate was prepared by treatment for 30 min at 95°C. The pyruvate assay kit (Cayman Chemicals) was used to measure intracellular pyruvate concentrations in the supernatant as described by the manufacturer.

### RNA isolation and sequencing.

For processing RNA from planktonic cultures, the ScriptSeq v2 transcriptome sequencing (RNA-Seq) library prep kit (Illumina) was used according to the vendor’s protocol. To isolate RNA from tumors, tumor-bearing mice were sacrificed 36 h postinfection. The tumor was squeezed twice through nylon filters (70 µm) and rinsed with RNAprotect solution. The suspension was centrifuged at 400 × *g* to settle cell debris. RNA extraction, cDNA preparation, and deep sequencing were performed as previously described (41).

### Quantification of gene expression.

Sequence reads were separated according to their bar codes mapped to the genome sequence of the reference strain *Salmonella enterica* subsp. *enterica* serovar Typhimurium UK-1 (GenBank accession no. CP002614.1) using Stampy (42). The R package DESeq (43) was used for differential gene expression analysis. Differentially expressed genes were identified using the *nbinomTest* function based on the negative binomial model. The Benjamini-Hochberg correction was used to control false discovery rate (FDR) at 5% in order to determine the list of regulated genes. Genes were identified as differentially expressed when they fulfilled the following criteria: (i) at least 2-fold down- or upregulation in comparison to the Wt and (ii) a Benjamini-Hochberg-corrected *P* value lower than 5%.

### Metabolic studies.

Bacteria were fed with 50 mg/liter [U-^13^C]glucose (99% ^13^C). Fatty acids and amino acids were analyzed after 1, 2, 4, and 8 h according to a published protocol (44). For a more detailed analysis of lipid metabolism, lipids were separated into neutral lipids, glycolipids, and phospholipids as described previously (45). The fractions were saponified and analyzed as described for the cellular fatty acids. Isotope ratio data are given in δ^13^C (‰). Controls followed the same protocol but used glucose with natural ^13^C abundance.

### Isolation of peptidoglycan and UPLC analysis.

Wt and mutant strains were harvested in stationary phase by centrifugation and quickly resuspended in 1× PBS buffer. Purification of peptidoglycan was performed as described previously (46) and analyzed by UPLC. Relative amounts of the muropeptides were calculated as described by Glauner (47).

### Complement sensitivity.

Human blood was taken from volunteers. Serum was isolated using Microvette serum tubes (Sarstedt). Bacteria were adjusted to 2 × 10^7^ CFU and challenged with serum by mixing it at 1:1. Heat-inactivated serum was prepared for 2 h at 56°C as a control. The reaction mixture was incubated for 30 min at 37°C. The remaining CFU were determined by plating.

### Invasion assay.

J774 cells were used to determine the phagocytic uptake and intracellular replication of the bacteria. The assay was performed as described previously using a multiplicity of infection (MOI) of 1 (48). Uptake was assayed 2 h postinfection, and intracellular replication was assayed 18 h postinfection. All values were compared to Wt.

### TNF-α measurement in serum.

Blood samples were collected 1.5 h postinfection. The TNF-α ELISA Max standard kit (BioLegend) was used to determine the TNF-α level in serum according to the manufacturer’s manual. Three biological replicates were analyzed, and a PBS-treated group served as negative control.

### Murine tumor model.

Six- to 7-week-old BALB/c mice (Janvier) were intradermally inoculated with 5 × 10^5^ syngeneic CT26 tumor cells (colorectal cancer, ATCC CRL-2638) or 2 × 10^6^ RenCa tumor cells (renal adenocarcinoma) in the right flank. The tumor establishment was monitored using a caliper. Upon reaching a tumor volume of approximately 150 mm³ after 10 days, the mice were injected intravenously in the tail vein with 5 × 10^6^ *Salmonella* bacteria.

### Therapeutic benefit and bacterial burden.

Tumor development was monitored with a caliper until tumors either were cleared or grew too large (>1,000 mm³). Body weight was monitored with a scale as a general health indicator. Mice were euthanized when the body weight dropped below 80% of initial weight at day 0 of infection.

### IFN-β reporter mice.

IFN-β^+/Δβ-luc^ reporter BALB/c mice (HZI) were used to measure endogenous IFN-β induction by *Salmonella* (21). Before imaging, 150 mg d-luciferin/kg of body weight was administered via intravenous injection. The mice were anesthetized with isoflurane (Baxter) and imaged using an IVIS 200 imaging system. Photon flux was quantified by Living Image 3.0 software (Caliper).

### Statistics.

Statistical analyses were performed using the two-tailed Student *t* test with *P* values of <0.05 considered significant.

### Accession number(s).

All raw and processed expression data have been submitted to GEO under accession number GSE74433.

## SUPPLEMENTAL MATERIAL

Figure S1  Colonization profile and cytokine measurement after infection with *Salmonella* variants. (A to D) Blood, spleen, liver, and tumor bacterial burdens were determined by plating serial dilutions of tissue homogenates. CFU counts of SF103 (Δ*lpxR9* Δ*pagL7* Δ*pagP8* Δ*rfaG*) and SF104 (Δ*lpxR9* Δ*pagL7* Δ*pagP8* Δ*aroA* Δ*rfaG*) at 12 hpi (A) and 36 hpi (B). CFU counts of Wt and SF101 (Δ*aroA*) at 12 hpi (C) and 36 hpi (D). (E) TNF-α levels in the sera of mice, 1.5 h after infection with Wt strain 14028 and its *aroA*-deficient variant. (F and G) Determination of IFN-β induction using IFN-β reporter mice 2 h and 4 h after infection with SF100 (Δ*lpxR9* Δ*pagL7* Δ*pagP8*) and SF102 (Δ*lpxR9* Δ*pagL7* Δ*pagP8* Δ*aroA*). The means and standard deviations are displayed. Results are representative for two independent experiments with 3 replicates per group. *, *P* < 0.05. Download Figure S1, PDF file, 0.4 MB

Figure S2  Characterization of *aroC* (SF137), *aroD* (SF138), and *aroC aroD* double mutant (SF139) strains. (A) MIC values for EDTA (millimolar) of Wt and *aro*-deficient strains SF137, SF138, and SF139. (B) Motility was assessed on semisolid agar. Means with standard deviations are displayed. Results are representative for two independent experiments with 5 biological replicates per group. Download Figure S2, PDF file, 0.2 MB

Figure S3  Electron microscopy and *in vitro* as well as *in vivo* complementation of *aroA*-deficient mutants SF101 and SF102. (A) Electron microscopy of negatively stained Wt and SF101 (Δ*aroA*) *Salmonella*. (B) Growth of SF102 (Δ*lpxR9* Δ*pagL7* Δ*pagP8* Δ*aroA*) or SF106 (Δ*lpxR9* Δ*pagL7* Δ*pagP8* Δ*aroA p-aroA*) in 1% (wt/vol) galactose minimal medium. (C) Motility assay. Motility of SF102 was restored by introducing the plasmid containing the gene *aroA* (SF106). (D) *In vitro* sensitivity toward human complement. Bacteria (2 × 10^7^) were incubated with either untreated or heat-inactivated (HI) human serum for 30 min at 37°C. The lysis effect was determined by plating. The increased susceptibility was abolished in the complemented strains SF105 (Δ*aroA p-aroA*) and SF106. (E) Body weight measurement as indicator for general health of mice infected with SF101 and SF102 and the complemented strains SF105 and SF106. The means with standard deviations are displayed. Results are representative for two independent experiments with 5 biological replicates per group. Download Figure S3, PDF file, 0.3 MB

Figure S4  Characterization of *ubiG* (SF140)- and *ubiA* (SF141)-deficient mutant strains. (A) MIC values for EDTA (millimolar) of Wt, SF101 (Δ*aroA*), and *ubi*-deficient strains SF140 and SF141. (B) Growth curve of the particular mutants compared to Wt. (C) TNF-α levels in the sera of mice, 1.5 h after infection with Wt and mutant strains SF101, SF140, and SF141. The means and standard deviations are displayed. Results are representative for two independent experiments with 4 replicates per group. Download Figure S4, PDF file, 0.2 MB

Figure S5  Metabolic and peptidoglycan analysis of *Salmonella* strains. (A) FAME analysis of Wt and SF101 (Δ*aroA*) to evaluate the fatty acid composition by gas chromatography. (B) FAME analysis of SF100 (Δ*lpxR9* Δ*pagL7* Δ*pagP8*) and SF102 (Δ*lpxR9* Δ*pagL7* Δ*pagP8* Δ*aroA*) to evaluate the fatty acid composition. (C) Differential turnover of fatty and amino acids in Wt and SF100. *Salmonella* bacteria were fed with ^13^C-labeled glucose, and the ^13^C/^12^C ratios of fatty acids were measured. Values of the Wt strain were subtracted from those of SF100. Positive values indicate that the metabolic turnover from ^13^C-labeled glucose to fatty acid was significantly higher than that for the Wt strain. In general, the lipid A modification already has an impact on the fatty acid metabolism. (D) ^13^C incorporation during amino acid metabolism. Analysis was carried out as in the experiment shown in panel A. (E) Ultraperformance liquid chromatography analysis of peptidoglycan from wild type (Wt), SF101 (Δ*aroA*), SF100 (Δ*lpxR9* Δ*pagL7* Δ*pagP8*), and SF102 (Δ*lpxR9* Δ*pagL7* Δ*pagP8* Δ*aroA*). Graphs were normalized by scaling the chromatograms relative to the maximum intensity measured in each run. The values are the means from two independent experiments. The means with standard deviations are displayed. Results are representative for two independent experiments with 3 biological replicates per group. *, *P* < 0.05. Download Figure S5, PDF file, 0.3 MB

Figure S6  Intracellular quantification of pyruvate and trehalose in *aro*-deficient mutants and their corresponding parental strains. (A) Strains were cultured overnight. Intracellular pyruvate levels of SF101 (Δ*aroA*), SF137 (Δ*aroC*), SF138 (Δ*aroD*), SF139 (Δ*aroD* Δ*aroC*), and SF102 (Δ*lpxR9* Δ*pagL7* Δ*pagP8* Δ*aroA*) were significantly increased. (B) All strains were cultured for 18 h at 37°C in LB medium. The trehalose levels were measured using the trehalose assay kit (Megazyme). The amounts of trehalose in the *aroA* mutants SF101 (Δ*aroA*) and SF102 (Δ*lpxR9* Δ*pagL7* Δ*pagP8* Δ*aroA*) were significantly smaller. The means with standard deviations are displayed. Results are representative for two independent experiments with 5 replicates per group. *, *P* < 0.05; **, *P* < 0.01. Download Figure S6, PDF file, 0.3 MB

Figure S7  *In vivo* transcriptome analysis of Wt and SF102 (Δ*lpxR9* Δ*pagL7* Δ*pagP8* Δ*aroA*) residing in CT26 tumors. Expression profile of the 20 most upregulated (top) and downregulated (bottom) genes in the *aroA*-deficient mutant SF102 in comparison to Wt. Depicted are normalized reads for every gene. Download Figure S7, PDF file, 0.5 MB

Table S1  Bacterial strains and plasmids used in this study. Table S1, PDF file, 0.2 MB

Table S2  *In vitro* transcriptome data of relevant genes upregulated and downregulated in SF102 (Δ*lpxR9* Δ*pagL7* Δ*pagP8* Δ*aroA*) in comparison to its parental strain SF100 (Δ*lpxR9* Δ*pagL7* Δ*pagP8*). Table S2, PDF file, 0.4 MB

Table S3  *In vivo* transcriptome data of relevant genes upregulated and downregulated in SF102 (Δ*lpxR9* Δ*pagL7* Δ*pagP8* Δ*aroA*) in comparison to Wt. Table S3, PDF file, 0.4 MB
