# Supplementary material for: aroA-Deficient Salmonella enterica Serovar Typhimurium Is More Than a Metabolically Attenuated Mutant
Source: mBio. 2016 Sep 6;7(5):e01220-16. doi: 10.1128/mBio.01220-16 (PMC5013297; doi:10.1128/mBio.01220-16)
Supplement: Figure S1 — Colonization profile and cytokine measurement after infection with Salmonella variants. (A to D) Blood, spleen, liver, and tumor bacterial burdens were determined by plating serial dilutions of tissue homogenates. CFU counts of SF103 (ΔlpxR9 ΔpagL7 ΔpagP8 ΔrfaG) and SF104 (ΔlpxR9 ΔpagL7 ΔpagP8 ΔaroA ΔrfaG) at 12 hpi (A) and 36 hpi (B). CFU counts of Wt and SF101 (ΔaroA) at 12 hpi (C) and 36 hpi (D). (E) TNF-α levels in the sera of mice, 1.5 h after infection with Wt strain 14028 and its aroA-deficient variant. (F and G) Determination of IFN-β induction using IFN-β reporter mice 2 h and 4 h after infection with SF100 (ΔlpxR9 ΔpagL7 ΔpagP8) and SF102 (ΔlpxR9 ΔpagL7 ΔpagP8 ΔaroA). The means and standard deviations are displayed. Results are representative for two independent experiments with 3 replicates per group. *, P < 0.05. Download [file mbo004162971sf1.pdf]

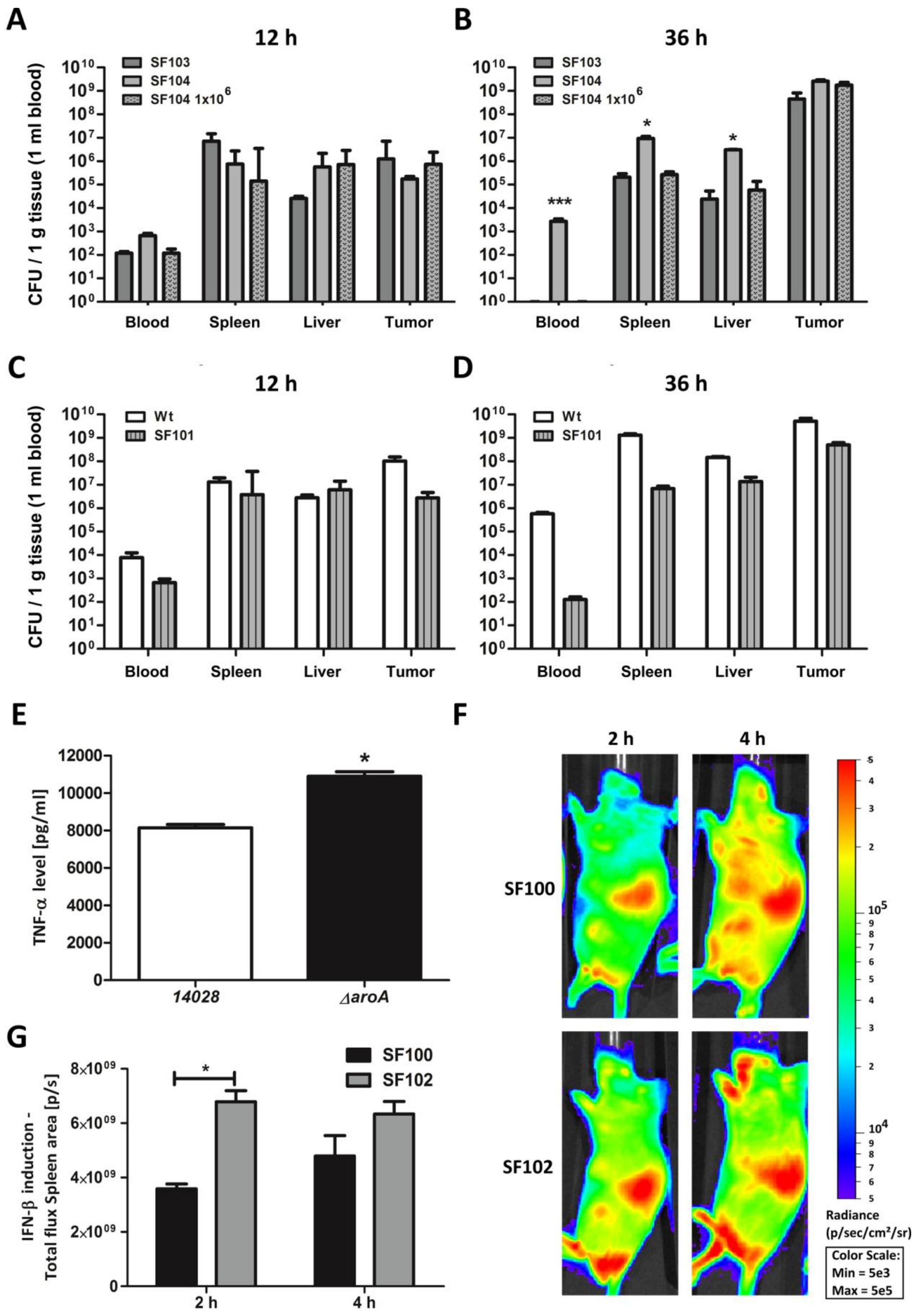


**Fig. S1. Colonization profile and cytokine measurement after infection with *Salmonella* variants.** (A-D) Blood, spleen, liver and tumor bacterial burdens were determined by plating serial dilutions of tissue homogenates. CFU counts of SF103 (Δ*lpxR9* Δ*pagL7* Δ*pagP8* Δ*rfaG*) and SF104 (Δ*lpxR9* Δ*pagL7* Δ*pagP8* Δ*aroA* Δ*rfaG*) at 12 hpi (A) and 36 hpi (B). CFU counts of Wt and SF101 (Δ*aroA*) at 12 hpi (C) and 36 hpi (D). (E) TNF-α level in the sera of mice, 1.5 h post infection with WT strain 14028 and its *aroA* deficient variant. (F) and (G) Determination of IFN-β induction using IFN-β reporter mice 2 h and 4 h post infection with SF100 (Δ*lpxR9* Δ*pagL7* Δ*pagP8*) and SF102 (Δ*lpxR9* Δ*pagL7* Δ*pagP8* Δ*aroA*). The mean and SDM is displayed. Results are representative for two independent experiments with 3 replicates per group. * $p<0.05$.
