# Supplementary material for: aroA-Deficient Salmonella enterica Serovar Typhimurium Is More Than a Metabolically Attenuated Mutant
Source: mBio. 2016 Sep 6;7(5):e01220-16. doi: 10.1128/mBio.01220-16 (PMC5013297; doi:10.1128/mBio.01220-16)
Supplement: Figure S2 — Characterization of aroC (SF137), aroD (SF138), and aroC aroD double mutant (SF139) strains. (A) MIC values for EDTA (millimolar) of Wt and aro-deficient strains SF137, SF138, and SF139. (B) Motility was assessed on semisolid agar. Means with standard deviations are displayed. Results are representative for two independent experiments with 5 biological replicates per group. Download [file mbo004162971sf2.pdf]

A

Strain	MIC_{EDTA}
WT	≈ 62
SF137	≈ 0.65
SF138	≈ 0.65
SF139	≈ 0.65

B

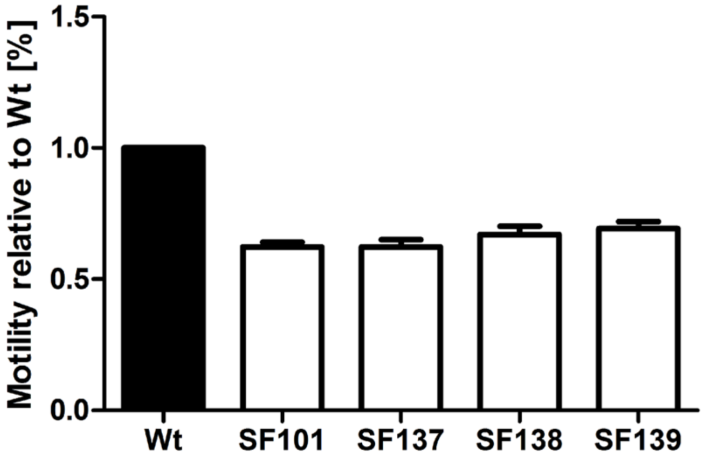

Fig. S2. Characterization of *aroC* (SF137), *aroD* (SF138) and *aroC aroD* double mutant (SF139). (A) MIC values for EDTA [mM] of WT and *aro* deficient strains SF137, SF138 and SF139. (B) The motility assessed on semi-solid agar. Mean with SDM is displayed. Results are representative for two independent experiments with 5 biological replicates per group.
