# Supplementary material for: aroA-Deficient Salmonella enterica Serovar Typhimurium Is More Than a Metabolically Attenuated Mutant
Source: mBio. 2016 Sep 6;7(5):e01220-16. doi: 10.1128/mBio.01220-16 (PMC5013297; doi:10.1128/mBio.01220-16)
Supplement: Figure S3 — Electron microscopy and in vitro as well as in vivo complementation of aroA-deficient mutants SF101 and SF102. (A) Electron microscopy of negatively stained Wt and SF101 (ΔaroA) Salmonella. (B) Growth of SF102 (ΔlpxR9 ΔpagL7 ΔpagP8 ΔaroA) or SF106 (ΔlpxR9 ΔpagL7 ΔpagP8 ΔaroA p-aroA) in 1% (wt/vol) galactose minimal medium. (C) Motility assay. Motility of SF102 was restored by introducing the plasmid containing the gene aroA (SF106). (D) In vitro sensitivity toward human complement. Bacteria (2 × 10^7^) were incubated with either untreated or heat-inactivated (HI) human serum for 30 min at 37°C. The lysis effect was determined by plating. The increased susceptibility was abolished in the complemented strains SF105 (ΔaroA p-aroA) and SF106. (E) Body weight measurement as indicator for general health of mice infected with SF101 and SF102 and the complemented strains SF105 and SF106. The means with standard deviations are displayed. Results are representative for two independent experiments with 5 biological replicates per group. Download [file mbo004162971sf3.pdf]

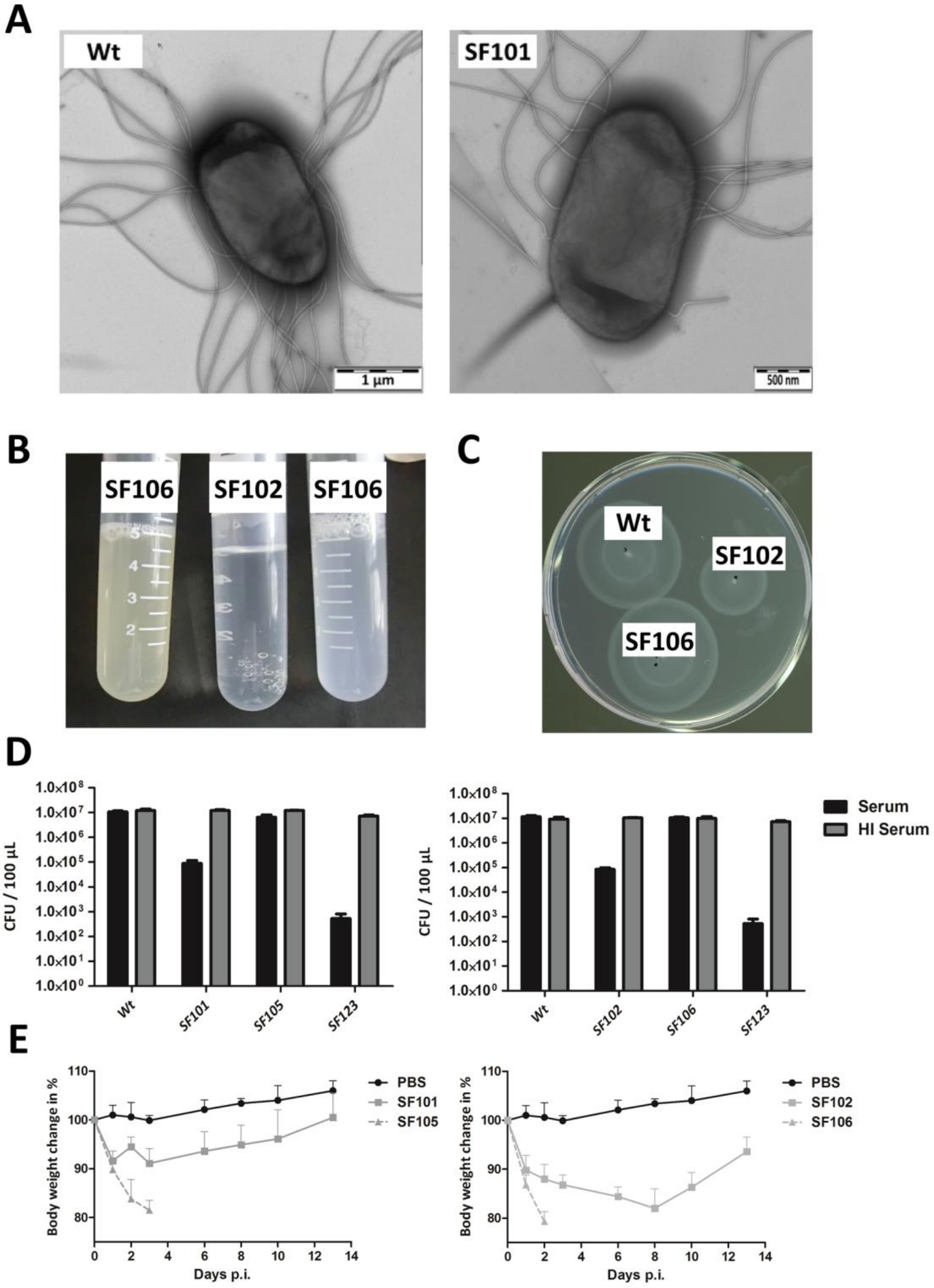

Fig. S3. Electron microscopy and *in vitro* as well as *in vivo* complementation of *aroA* deficient mutants SF101 and SF102. (A) Electron microscopy of negatively stained of Wt and SF101 (Δ*aroA*) *Salmonella*. (B) Growth of SF102 (Δ*lpxR9*, Δ*pagL7*, Δ*pagP8* Δ*aroA*) or SF106 (Δ*lpxR9*, Δ*pagL7*, Δ*pagP8* Δ*aroA p-aroA*) in 1% (w/v) galactose minimal media. (C) Motility assay. Motility of SF102 was restored by introducing the plasmid containing the gene *aroA* (SF106). (D) *In-vitro* sensitivity towards human complement. $2x10^7$ bacteria were incubated with either untreated or heat inactivated (HI) human serum for 30 min at 37°C. The lysis effect was determined by plating. The increased susceptibility was abolished in the complemented strains SF105 (Δ*aroA p-aroA*) and SF106. (E) Body weight measurement as

indicator for general health of mice infected with SF101 and SF102 and the complemented strains SF105 and SF106. The mean with standard deviation is displayed. Results are representative for two independent experiments with 5 biological replicates per group.
