# Supplementary material for: aroA-Deficient Salmonella enterica Serovar Typhimurium Is More Than a Metabolically Attenuated Mutant
Source: mBio. 2016 Sep 6;7(5):e01220-16. doi: 10.1128/mBio.01220-16 (PMC5013297; doi:10.1128/mBio.01220-16)
Supplement: Figure S4 — Characterization of ubiG (SF140)- and ubiA (SF141)-deficient mutant strains. (A) MIC values for EDTA (millimolar) of Wt, SF101 (ΔaroA), and ubi-deficient strains SF140 and SF141. (B) Growth curve of the particular mutants compared to Wt. (C) TNF-α levels in the sera of mice, 1.5 h after infection with Wt and mutant strains SF101, SF140, and SF141. The means and standard deviations are displayed. Results are representative for two independent experiments with 4 replicates per group. Download [file mbo004162971sf4.pdf]

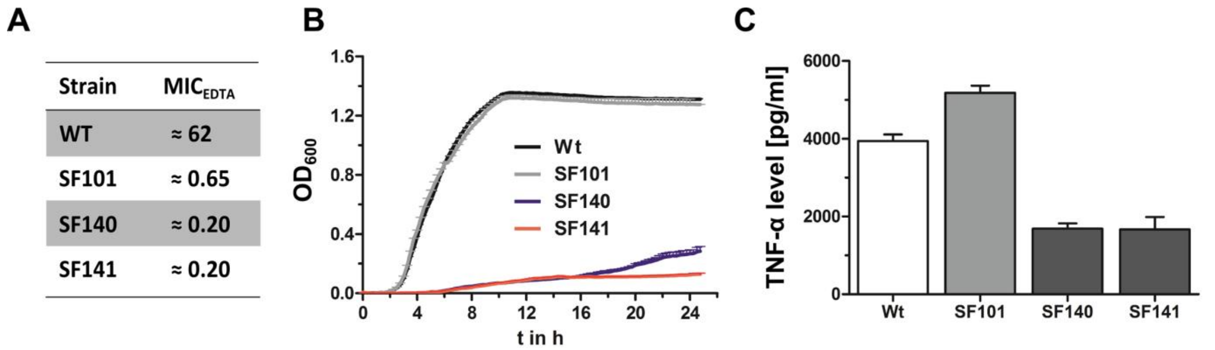

Fig. S4. Characterization of *ubiG* (SF140) and *ubiA* (SF141) deficient mutant strains. (A) MIC values for EDTA [mM] of WT, SF101 (Δ*aroA*) and *ubi* deficient strains SF140 and SF141. (B) Growth curve of the particular mutants compared to Wt. (C) TNF-α level in the sera of mice, 1.5 h post infection with WT and mutant strains SF101, SF140 and SF141. The mean and SDM is displayed. Results are representative for two independent experiments with 4 replicates per group.
