# Supplementary material for: aroA-Deficient Salmonella enterica Serovar Typhimurium Is More Than a Metabolically Attenuated Mutant
Source: mBio. 2016 Sep 6;7(5):e01220-16. doi: 10.1128/mBio.01220-16 (PMC5013297; doi:10.1128/mBio.01220-16)
Supplement: Figure S5 — Metabolic and peptidoglycan analysis of Salmonella strains. (A) FAME analysis of Wt and SF101 (ΔaroA) to evaluate the fatty acid composition by gas chromatography. (B) FAME analysis of SF100 (ΔlpxR9 ΔpagL7 ΔpagP8) and SF102 (ΔlpxR9 ΔpagL7 ΔpagP8 ΔaroA) to evaluate the fatty acid composition. (C) Differential turnover of fatty and amino acids in Wt and SF100. Salmonella bacteria were fed with 13C-labeled glucose, and the 13C/12C ratios of fatty acids were measured. Values of the Wt strain were subtracted from those of SF100. Positive values indicate that the metabolic turnover from 13C-labeled glucose to fatty acid was significantly higher than that for the Wt strain. In general, the lipid A modification already has an impact on the fatty acid metabolism. (D) 13C incorporation during amino acid metabolism. Analysis was carried out as in the experiment shown in panel A. (E) Ultraperformance liquid chromatography analysis of peptidoglycan from wild type (Wt), SF101 (ΔaroA), SF100 (ΔlpxR9 ΔpagL7 ΔpagP8), and SF102 (ΔlpxR9 ΔpagL7 ΔpagP8 ΔaroA). Graphs were normalized by scaling the chromatograms relative to the maximum intensity measured in each run. The values are the means from two independent experiments. The means with standard deviations are displayed. Results are representative for two independent experiments with 3 biological replicates per group. *, P < 0.05. Download [file mbo004162971sf5.pdf]

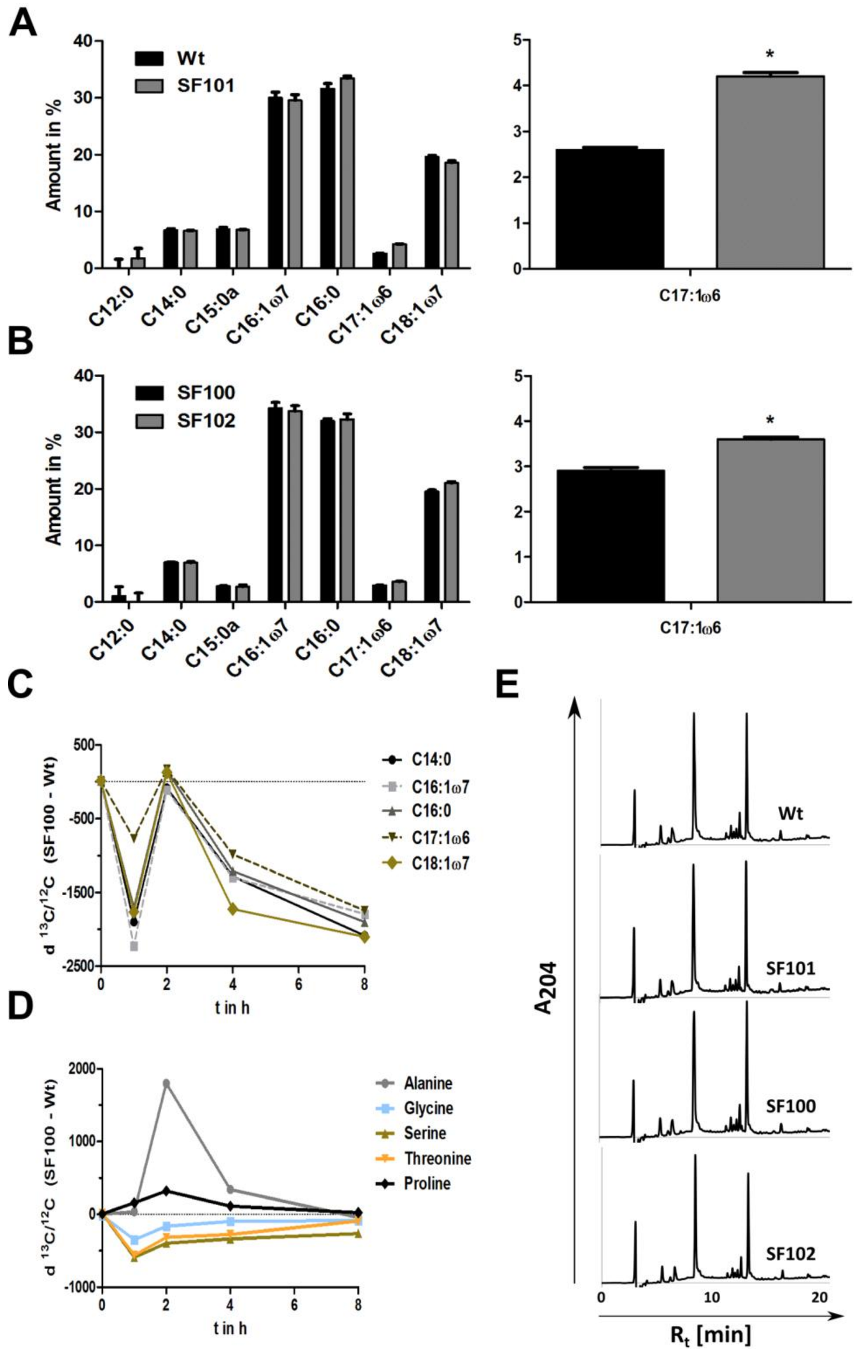

Fig. S5. Metabolic and peptidoglycan analysis of *Salmonella* strains. (A) FAME analysis of Wt and SF101 (Δ*aroA*) to evaluate the fatty acid composition by gas chromatography. (B) FAME analysis of SF100 (Δ*lpxR9* Δ*pagL7* Δ*pagP8*) and SF102 (Δ*lpxR9* Δ*pagL7* Δ*pagP8* Δ*aroA*) to evaluate the fatty acid composition. (C) Differential turnover of fatty and amino acids in Wt and SF100. *Salmonella* were fed with ^{13}C labeled glucose and the $^{13}C/^{12}C$ ratios of fatty acids were measured. Values of the Wt strain were subtracted from those of SF100. Positive values indicate that the metabolic turnover from ^{13}C labeled glucose to fatty acid was significantly higher than for the Wt strain. In general, the Lipid A modification already has an impact on the fatty acid metabolism. (D) ^{13}C incorporation during amino acid metabolism.

Analysis was carried out as in A. (E) Ultra performance liquid chromatography analysis of PG from wild-type (Wt), SF101 (Δ*aroA*), SF100 (Δ*lpxR9* Δ*pagL7* Δ*pagP8*) and SF102 (Δ*lpxR9* Δ*pagL7* Δ*pagP8* Δ*aroA*). Graphs were normalized by scaling the chromatograms relative to the maximum intensity measured in each run. The mean with standard deviation is displayed. Results are representative for two independent experiments with 3 biological replicates per group. * $p<0.05$.
