# Supplementary material for: aroA-Deficient Salmonella enterica Serovar Typhimurium Is More Than a Metabolically Attenuated Mutant
Source: mBio. 2016 Sep 6;7(5):e01220-16. doi: 10.1128/mBio.01220-16 (PMC5013297; doi:10.1128/mBio.01220-16)
Supplement: Figure S6 — Intracellular quantification of pyruvate and trehalose in aro-deficient mutants and their corresponding parental strains. (A) Strains were cultured overnight. Intracellular pyruvate levels of SF101 (ΔaroA), SF137 (ΔaroC), SF138 (ΔaroD), SF139 (ΔaroD ΔaroC), and SF102 (ΔlpxR9 ΔpagL7 ΔpagP8 ΔaroA) were significantly increased. (B) All strains were cultured for 18 h at 37°C in LB medium. The trehalose levels were measured using the trehalose assay kit (Megazyme). The amounts of trehalose in the aroA mutants SF101 (ΔaroA) and SF102 (ΔlpxR9 ΔpagL7 ΔpagP8 ΔaroA) were significantly smaller. The means with standard deviations are displayed. Results are representative for two independent experiments with 5 replicates per group. *, P < 0.05; **, P < 0.01. Download [file mbo004162971sf6.pdf]

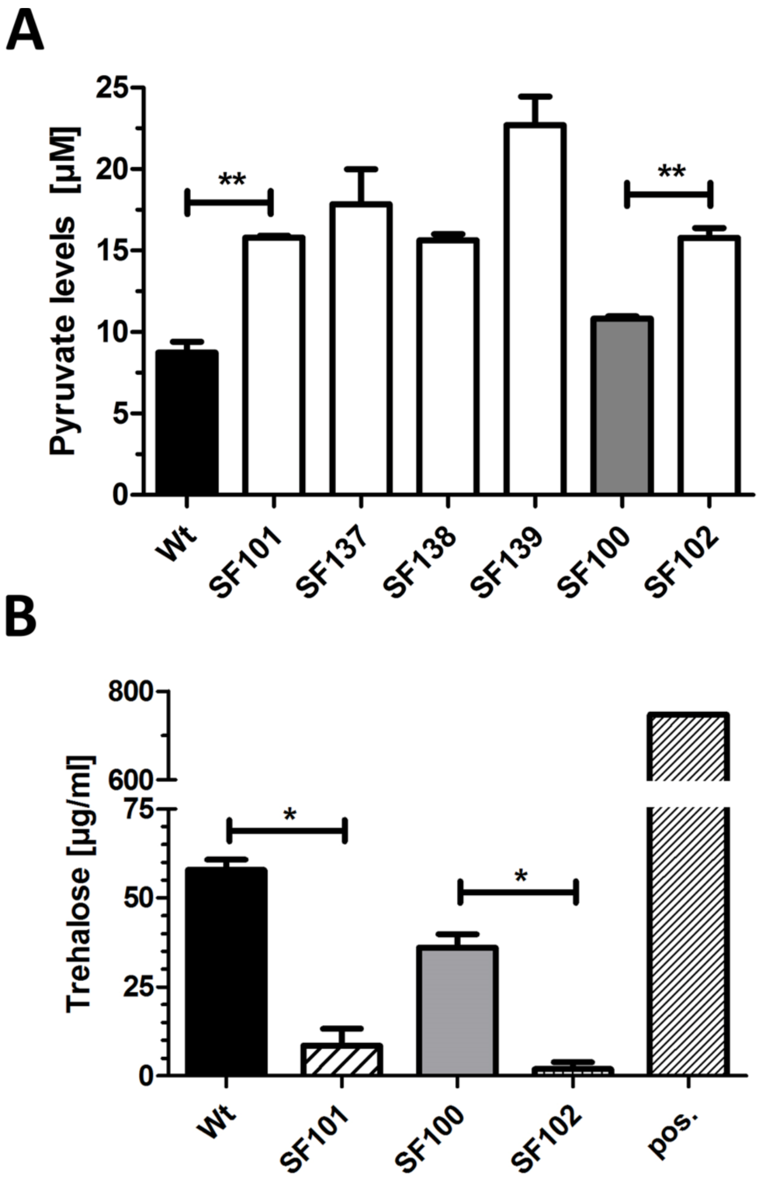

Fig. S6. Intracellular quantification of pyruvate and trehalose in *aro* deficient mutants and their corresponding parental strains. (A) Strains were cultured over night. Intracellulare pyruvate levels of SF101 (Δ*aroA*), SF137 (Δ*aroC*), SF138 (Δ*aroD*) SF139 (Δ*aroD* Δ*aroC*) and SF102 (Δ*lpxR9* Δ*pagL7* Δ*pagP8* Δ*aroA*) were significantly increased. (B) All strains were cultured for 18 h at 37°C in LB media. The trehalose levels were measured using the Trehalose Assay Kit (Megazym). The amount of trehalose in the *aroA* mutants SF101 (Δ*aroA*) and SF102 (Δ*lpxR9* Δ*pagL7* Δ*pagP8* Δ*aroA*) were significantly lower. The mean with standard deviation is displayed. Results are representative for two independent experiments with 5 replicates per group. * $p<0.05$, ** $p<0.01$.
