# Supplementary material for: aroA-Deficient Salmonella enterica Serovar Typhimurium Is More Than a Metabolically Attenuated Mutant
Source: mBio. 2016 Sep 6;7(5):e01220-16. doi: 10.1128/mBio.01220-16 (PMC5013297; doi:10.1128/mBio.01220-16)
Supplement: Figure S7 — In vivo transcriptome analysis of Wt and SF102 (ΔlpxR9 ΔpagL7 ΔpagP8 ΔaroA) residing in CT26 tumors. Expression profile of the 20 most upregulated (top) and downregulated (bottom) genes in the aroA-deficient mutant SF102 in comparison to Wt. Depicted are normalized reads for every gene. Download [file mbo004162971sf7.pdf]

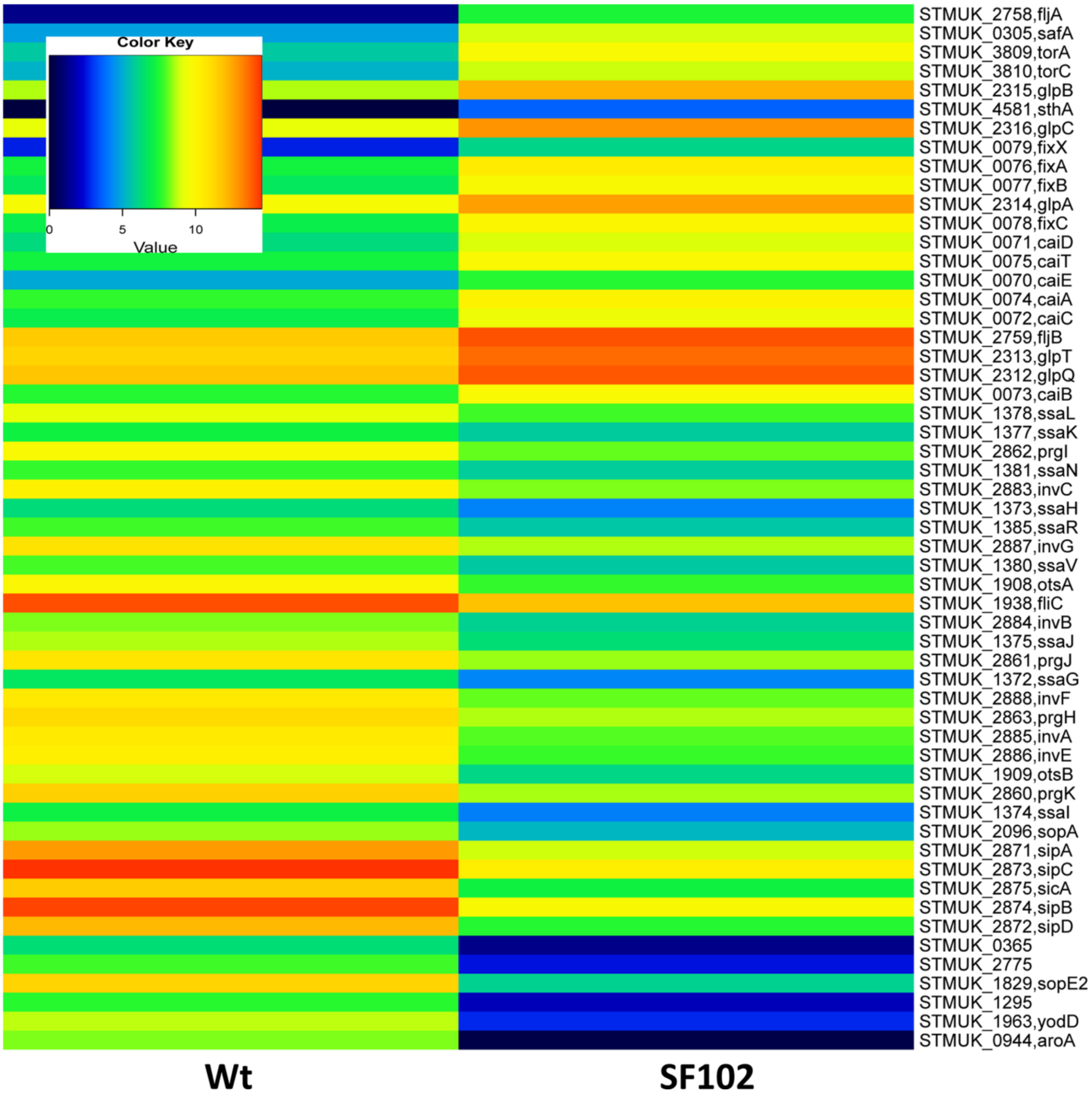

Fig. S7 *In vivo* transcriptome analysis of Wt and SF102 (Δ*lpxR9* Δ*pagL7* Δ*pagP8* Δ*aroA*) residing in CT26 tumors. Expression profile of the twenty most upregulated (top) and downregulated (bottom) genes in the *aroA* deficient mutant SF102 in comparison to Wt. Depicted are fold changes for every gene.
