# Supplementary material for: aroA-Deficient Salmonella enterica Serovar Typhimurium Is More Than a Metabolically Attenuated Mutant
Source: mBio. 2016 Sep 6;7(5):e01220-16. doi: 10.1128/mBio.01220-16 (PMC5013297; doi:10.1128/mBio.01220-16)
Supplement: Table S1 — Bacterial strains and plasmids used in this study. [file mbo004162971st1.pdf]

Tab. S1: Bacterial strains and plasmids used in this study

Strain	Description	Source	Ref.
**Parental strains**			
**Wt**	Wild-type	**χ3761**	-
**SF100**	*ΔlpxR9 ΔpagL7 ΔpagP8*	**χ9485**	Kong et al. 2011
***aroA* mutants**			
**SF101**	*ΔaroA*	This study	-
**SF102**	*ΔlpxR9 ΔpagL7 ΔpagP8 ΔaroA*	This study	-
**SF103**	*ΔlpxR9 ΔpagL7 ΔpagP8 ΔrfaG42*	This study	-
**SF104**	*ΔlpxR9 ΔpagL7 ΔpagP8 ΔaroA ΔrfaG42*	This study	-
**SF105**	*ΔaroA p-aroA*	This study	-
**SF106**	*ΔlpxR9, ΔpagL7, ΔpagP8 ΔaroA p-aroA*	This study	-
**SF137**	*ΔaroC1083*	**χ9954**	Curtiss Lab
**SF138**	*ΔaroD769*	**χ9956**	Curtiss Lab
**SF139**	*ΔaroD769 ΔaroC1083*	This study	-
**Flagella mutants**			
**SF109**	*flhDC5213::MudJ* (Class I)	This study	-
**SF110**	*fliL5100::MudJ* (Class II)	This study	-
**SF111**	*fljB5001::MudJ Δhin::FRT* (Class III)	This study	-
**SF112**	SF101 + *flhDC5213::MudJ* (Class I)	This study	-
**SF113**	SF101+ *fliL5100::MudJ* (Class II)	This study	-
**SF114**	SF101 + *fljB5001::MudJ Δhin::FRT* (Class III)	This study	-
**SF115**	SF100 + *flhDC5213::MudJ* (Class I)	This study	-
**SF116**	SF100+ *fliL5100::MudJ* (Class II)	This study	-
**SF117**	SF100 + *fljB5001::MudJ Δhin::FRT* (Class III)	This study	-
**SF118**	SF102 + *flhDC5213::MudJ* (Class I)	This study	-
**SF119**	SF102 + *fliL5100::MudJ* (Class II)	This study	-
**SF120**	SF102 + *fljB5001::MudJ Δhin::FRT* (Class III)	This study	-
**SF121**	*Δhin::FCF* ($FljB^{ON}$)	This study	-
**SF122**	*ΔfliC::FCF*	This study	-
**SF123**	SF101 + *Δhin::FCF* ($FljB^{ON}$)	This study	-
**SF124**	SF101 + *ΔfliC::FCF*	This study	-
**SF125**	SF100 + *Δhin::FCF* ($FljB^{ON}$)	This study	-
**SF126**	SF100 + *ΔfliC::FCF*	This study	-
**SF127**	SF102 + *Δhin::FCF* ($FljB^{ON}$)	This study	-
**SF128**	SF102 + *ΔfliC::FCF*	This study	-
**SF129**	*fliC::MudJ*	This study	-
**SF130**	SF101 + *fliC::MudJ*	This study	-
**SF131**	SF100 + *fliC::MudJ*	This study	-
**SF132**	SF102 + *fliC::MudJ*	This study	-
**LPS mutants**			
**SF133**	*ΔarnT::* FKF	This study	-
**SF134**	*ΔlpxR9 ΔpagL7 ΔpagP8 ΔaroA ΔarnT::* FKF	This study	-
**SF135**	*ΔrfaG*	This study	-

**SF136**	*ΔrfaG ΔaroA*	This study	-
**Other mutants**			
**SF140**	*ΔubiG::* FKF	This study	-
**SF141**	*ΔubiA::* FKF	This study	-
**SF142**	*ΔansB::* FKF	This study	-
**SF143**	SF101 + *ΔansB::FKF*	This study	-
**Plamids**			
**pYA3600**	Suicide vector *aroA*	R. Curtiss	Curtiss Lab
**pYA4896**	Suicide vector *rfaG*	R. Curtiss	Kong et al. 2011
**pTrc99A**	*lac* pr, *ori* , Amp^R	M. Erhardt	-
