# Supplementary material for: aroA-Deficient Salmonella enterica Serovar Typhimurium Is More Than a Metabolically Attenuated Mutant
Source: mBio. 2016 Sep 6;7(5):e01220-16. doi: 10.1128/mBio.01220-16 (PMC5013297; doi:10.1128/mBio.01220-16)
Supplement: Table S2 — In vitro transcriptome data of relevant genes upregulated and downregulated in SF102 (ΔlpxR9 ΔpagL7 ΔpagP8 ΔaroA) in comparison to its parental strain SF100 (ΔlpxR9 ΔpagL7 ΔpagP8). [file mbo004162971st2.pdf]

Tab. S2: *In vitro* transcriptome data of relevant genes upregulated and downregulated in SF102 (*Δ lpxR9 ΔpagL7 ΔpagP8 ΔaroA*) in comparison to its parental strain SF100 (*ΔlpxR9 ΔpagL7 ΔpagP8*).

Gene	Description	Log2FC
**SF102 - upregulated**		
STMUK_2758,*fljA*	filament fljB	3,92
STMUK_2473,*cysA*	sulfate/thiosulfate transporter	3,45
STMUK_2475,*cysU*	sulfate/thiosulfate transporter	2,83
STMUK_2759,*fljB*	filament fljB	2,72
STMUK_2474,*cysW*	sulfate/thiosulfate transporter	2,59
STMUK_2936,*cysI*	sulfite reductase subunit beta	2,52
STMUK_1947,fliE	basal body	2,5
STMUK_3094,*ansB*	L-asparaginase II	2,47
STMUK_2907,*ygbJ*	3-hydroxyisobutyrate dehydrogenase	2,45
STMUK_1142,*flgB*	basal body	2,41
STMUK_1143,*flgC*	basal body	2,23
STMUK_2935,*cysH*	phosphoadenosine phosphosulfate reductase	2,23
STMUK_1221,	putative outer membrane lipoprotein	2,17
STMUK_1144,*flgD*	basal body	2,15
STMUK_2903,	putative nucleoside-diphosphate-sugar epimerase	2,15
STMUK_1145,*flgE*	basal body	2,14
STMUK_2313,*glpT*	sn-glycerol-3-phosphate transporter	2,07
STMUK_3064,*tktA*	transketolase	2,03
STMUK_1147,*flgG*	basal body	1,96
STMUK_3599,*dctA*	C4-dicarboxylate transporter DctA	1,93
STMUK_2959,*sdaB*	L-serine dehydratase/L-threonine deaminase	1,85
STMUK_2312,*glpQ*	glycerophosphodiester phosphodiesterase	1,6
**SF102 - downregulated**		
STMUK_1804,*manY*	mannose-specific enzyme IIC	-1,21
STMUK_1805,*manZ*	PTS system mannose-specific transporter subunit IID	-1,25
STMUK_1803,*manX*	mannose-specific enzyme IIAB	-1,45
STMUK_1908,*otsA*	trehalose-6-phosphate phosphatase	-1,45
STMUK_2195,*yehZ*	osmoprotectant transport system substrate-binding protein	-1,87
STMUK_2760,*hin*	flagella phase variator	-1,89
STMUK_2505,*talA*	transaldolase A	-1,93
STMUK_1278,*osmE*	DNA-binding transcriptional activator OsmE	-1,96
STMUK_1339,*sufS*	cysteine desulfurase activator complex subunit SufB	-1,99
STMUK_1909,*otsB*	trehalose-6-phosphate phosphatase	-2,01
STMUK_3595,*yhjG*	putative inner membrane protein	-2,11
STMUK_1372,*ssaG*	type III secretion system apparatus protein	-2,16
STMUK_3606,*yhjR*	putative cytoplasmic protein	-2,2
STMUK_0903,*poxB*	pyruvate dehydrogenase	-2,25
STMUK_4321,*ecnB*	entericidin B membrane lipoprotein	-2,45
STMUK_1338,*sufD*	cysteine desulfurase activator complex subunit SufB	-2,48
STMUK_1337,*sufC*	cysteine desulfurase activator complex subunit SufB	-2,61
STMUK_1449,*ynfM*	putative membrane transport protein	-2,82
STMUK_0877,*ybjM*	putative inner membrane protein	-2,87
STMUK_0116,*leuL*	Leucin operon leader peptide	-3,02
STMUK_3429,*bfr*	bacterioferritin	-3,07

STMUK_0171,*gcd*	glucose dehydrogenase	-3,09
STMUK_1938,*fliC*	filament	-3,14
STMUK_2170,*fbaB*	fructose-bisphosphate aldolase	-3,14
STMUK_1336,*sufB*	cysteine desulfurase activator complex subunit SufB	-3,17
STMUK_1533,*yddX*	biofilm-dependent modulation protein	-3,63
STMUK_4324,*blc*	outer membrane lipoprotein Blc	-3,63
STMUK_1335,*sufA*	iron-sulfur cluster assembly scaffold protein	-3,82
STMUK_0944,*aroA*	3-phosphoshikimate 1-carboxyvinyltransferase	-9,42
