# Supplementary material for: aroA-Deficient Salmonella enterica Serovar Typhimurium Is More Than a Metabolically Attenuated Mutant
Source: mBio. 2016 Sep 6;7(5):e01220-16. doi: 10.1128/mBio.01220-16 (PMC5013297; doi:10.1128/mBio.01220-16)
Supplement: Table S3 — In vivo transcriptome data of relevant genes upregulated and downregulated in SF102 (ΔlpxR9 ΔpagL7 ΔpagP8 ΔaroA) in comparison to Wt. [file mbo004162971st3.pdf]

Tab. S3: *In vivo* transcriptome data of relevant genes upregulated and downregulated in SF102 (*Δ lpxR9 ΔpagL7 ΔpagP8 ΔaroA*) in comparison to Wt.

Gene	Description	Log2FC
**SF102 - upregulated**		
STMUK_2758,*fljA*	phase-1 flagellin repressor	6,19
STMUK_0305,*safA*	putative outer membrane protein	4,21
STMUK_3809,*torA*	trimethylamine N-oxide reductase subunit	4,08
STMUK_3810,*torC*	trimethylamine N-oxide reductase cytochrome c-like subunit	3,72
STMUK_2315,*glpB*	anaerobic glycerol-3-phosphate dehydrogenase subunit B	3,64
STMUK_4581,*sthA*	sn-glycerol-3-phosphate dehydrogenase subunit C	3,64
STMUK_2316,*glpC*	putative ferredoxin	3,62
STMUK_0079,*fixX*	putative electron transfer flavoprotein FixA	3,4
STMUK_0076,*fixA*	putative electron transfer flavoprotein FixB	3,27
STMUK_0077,*fixB*	sn-glycerol-3-phosphate dehydrogenase subunit A	3,09
STMUK_2314,*glpA*	putative oxidoreductase FixC	3,08
STMUK_0071,*caiD*	phase-1 flagellin repressor	2,79
STMUK_0075,*caiT*	L-carnitine/gamma-butyrobetaine antiporter	2,61
STMUK_0070,*caiE*	carnitine operon protein CaiE	2,60
STMUK_0074,*caiA*	crotonobetainyl-CoA dehydrogenase	2,49
STMUK_0072,*caiC*	putative crotonobetaine/carnitine-CoA ligase	2,41
STMUK_2759,*fljB*	flagellin	2,38
STMUK_2313,*glpT*	sn-glycerol-3-phosphate transporter	2,27
STMUK_2312,*glpQ*	glycerophosphodiester phosphodiesterase	2,23
**SF102 - downregulated**		
STMUK_0633,*pagP*	palmitoyl transferase	-999,99
STMUK_0944,*aroA*	3-phosphoshikimate 1-carboxyvinyltransferase	-8,06
STMUK_1295,*pagL*	putative outer membrane protein	-5,67
STMUK_1829,*sopE2*	type III-secreted effector protein	-5,42
STMUK_2775,*lpxR*	tricarboxylic transport	-5,33
STMUK_2872,*sipD*	translocation machinery component	-4,57
STMUK_2874,*sipB*	translocation machinery component	-4,45
STMUK_2875,*sicA*	secretion chaperone	-4,36
STMUK_2873,*sipC*	translocation machinery component	-4,14
STMUK_2871,*sipA*	secreted effector protein	-3,89
STMUK_2096,*sopA*	secreted effector protein	-3,20
STMUK_1374,*ssaI*	type III secretion system apparatus protein	-2,99
STMUK_2860,*prgK*	needle complex inner membrane lipoprotein	-2,93
STMUK_1909,*otsB*	trehalose-6-phosphate phosphatase	-2,81
STMUK_2886,*invE*	invasion protein	-2,62
STMUK_2885,*invA*	needle complex export protein	-2,56
STMUK_2863,*prgH*	needle complex inner membrane protein	-2,52
STMUK_2888,*invF*	invasion regulatory protein	-2,49
STMUK_1372,*ssaG*	type III secretion system apparatus protein	-2,43
STMUK_2861,*prgJ*	needle complex minor subunit	-2,36
STMUK_1375,*ssaJ*	needle complex inner membrane lipoprotein	-2,32
STMUK_2884,*invB*	secretion chaperone	-2,3
STMUK_1938,*fliC*	flagellin	-2,23
STMUK_1908,*otsA*	trehalose-6-phosphate synthase	-2,22

STMUK_1380,*ssaV*	secretion system apparatus protein SsaV	-2,19
STMUK_2887,*invG*	outer membrane secretin precursor	-2,18
STMUK_1385,*ssaR*	type III secretion system protein	-2,17
STMUK_1373,*ssaH*	type III secretion system apparatus protein	-2,10
STMUK_2883,*invC*	ATP synthase SpaL	-1,97
STMUK_1381,*ssaN*	type III secretion system ATPase	-1,88
STMUK_2862,*prgI*	needle complex major subunit	-1,64
STMUK_2331,*arnT*	4-amino-4-deoxy-L-arabinose transferase	-1,52
STMUK_1377,*ssaK*	type III secretion system apparatus protein	-1,43
STMUK_1378,*ssaL*	type III secretion system apparatus protein	-1,42
